# Mesenchymal stem cell‐conditioned medium attenuates the retinal pathology in amyloid‐β‐induced rat model of Alzheimer's disease: Underlying mechanisms

**DOI:** 10.1111/acel.13340

**Published:** 2021-03-30

**Authors:** Shu‐Chun Kuo, Chung‐Ching Chio, Chao‐Hung Yeh, Jui‐Ti Ma, Wen‐Pin Liu, Mao‐Tsun Lin, Kao‐Chang Lin, Ching‐Ping Chang

**Affiliations:** ^1^ Department of Ophthalmology Chi Mei Medical Center Tainan Taiwan; ^2^ Department of Optometry Chung Hwa University of Medical Technology Tainan Taiwan; ^3^ Division of Neurosurgery Department of Surgery Chi Mei Medical Center Tainan Taiwan; ^4^ Department of Medical Research Chi Mei Medical Center Tainan Taiwan; ^5^ Department of Holistic Care Chi Mei Medical Center Tainan Taiwan; ^6^ Department of Neurology Chi Mei Medical Center Tainan Taiwan

**Keywords:** Alzheimer's disease, amyloid‐beta, retina pigment epithelium, secretome, stem cell

## Abstract

Amyloid‐beta (Aβ) oligomer is known to contribute to the pathophysiology of age‐related macular degeneration. Herein, we aimed to elucidate the *in vivo* and *in vitro* effects of Aβ_1‐42_ application on retinal morphology in rats. Our *in vivo* studies revealed that intracerebroventricular administration of Aβ_1‐42_ oligomer caused dysmorphological changes in both retinal ganglion cells and retinal pigment epithelium. In addition, *in vitro* studies revealed that ARPE‐19 cells following Aβ_1‐42_ oligomer application had decreased viability along with apoptosis and decreased expression of the tight junction proteins, increased expression of both phosphor‐AKT and phosphor‐GSK3β and decreased expression of both SIRT1 and β‐catenin. Application of conditioned medium (CM) obtained from mesenchymal stem cells (MSC) protected against Aβ_1‐42_ oligomer‐induced retinal pathology in both rats and ARPE‐19 cells. In order to explore the potential role of peptides secreted from the MSCs, we applied mass spectrometry to compare the peptidomics profiles of the MSC‐CM. Gene ontology enrichment analysis and String analysis were performed to explore the differentially expressed peptides by predicting the functions of their precursor proteins. Bioinformatics analysis showed that 3‐8 out of 155–163 proteins in the MSC‐CM maybe associated with SIRT1/pAKT/pGSK3β/β‐catenin, tight junction proteins, and apoptosis pathway. In particular, the secretomes information on the MSC‐CM may be helpful for the prevention and treatment of retinal pathology in age‐related macular degeneration.

## Graphical abstract

Aβ‐induced cell apoptosis may through inhibits SIRT1, enhanced AKT and GSK3β phosphorylation, and suppressed β‐catenin expression, and disrupted tight junction protein expression. It is well known that β‐catenin could maintain tight junction protein (such as ZO‐1 and occludin) expression and inhibit apoptosis. Conditioned medium (N‐CM or H‐CM) from mesenchymal stem cells under normoxic or hypoxic culture environment could reverse the Aβ‐induced apoptosis and tight junction protein disruption via APOC3, APOA1, KNG1, PSAP, HSP90B1, ACLY, YWHAE, and H2AFX from N‐CM, and CD44, PSMA1, and CTTN from H‐CM, which are all related to SIRT1/pAKT/pGSK3β/β‐catenin signaling pathway.

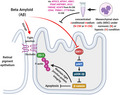

**Abbreviations**
AKT: a serine/threonine‐specific protein kinase
Aβ: amyloid‐beta
DAPI: 4’,6‐diamidino‐2‐phenylindole
GCL: ganglion cell layer
GSK3β: glycogen synthase kinase 3β
H‐CM: hypoxia mesenchymal stem cell‐conditioned medium
i.c.v.: intracerebroventricular
INL: inner nuclear layer
IPL: inner plexiform layer
IS: inner segments
MSC: mesenchymal stem cell
NA: numerical aperture
N‐CM: normoxia mesenchymal stem cell‐conditioned medium
NeuN: neuronal nuclei
NFL: nerve fiber layer
ONL: outer nuclear layer
OPL: outer plexiform layer
OS: outer segments
PI3K: phosphoinositide 3‐kinase
RPE: retinal pigment epithelium
SIRT1: sirtuin 1
TJP: tight junction protein
TUNEL: terminal deoxyribonucleotide transferase‐mediated dUTP nick end labeling
Veh: vehicle
ZO‐1: zonula occludens‐1

## 1 INTRODUCTION

The retina and optic nerve have similar patterns of vascularization and blood–tissue barrier function with brain (Kusne et al., 2017). Because of these similarities, the retina has been considered to be a source of biomarkers for Alzheimer's disease (AD) (Colligris et al., 2018). Retinal changes in AD include the followings: (a) reduction in the number of retinal ganglion cells (RGC); (b) decreased thickness in the retinal nerve fiber layer (NFL); (c) decreased choroidal thickness in the foveal area; (d) visual field reduction; and (e) accumulation of tau and amyloid‐beta (Aβ) (Abulfadl et al., 2018). Aβ observed in the brain of Alzheimer's disease (AD) patients share also been seen in the retinas of patients with retinal neurodegenerative conditions, such as age‐related macular degeneration (AMD) the retina has been considered to be a source of biomarkers for AD diagnosis. Dysfunction of retina pigment epithelial (RPE) cells is a significant risk factor for the development of AMD. Drusen or choroidal neovascularization (CNV) is the typical pathological features of AMD. A further experimental study indicated that SIRT1 levels were reduced in human RPE cells after treatment with Aβ, which is one of the constituents of drusen (Cao et al., 2013). Regarding retinal accumulation of Aβ in AD, the update evidence is equivocal. For example, a single intravitreal injection of the oligomeric of Aβ_1‐42_ has been shown to be toxic to the neural retina of rats (Walsh et al., 2005). Both *in vitro* and *in vivo* studies found that Aβ_1‐42_ reduced mitochondrial redox potential and increased the production of reactive oxygen species, but did not induce apoptosis in RPE cell cultures (Bruban et al., 2009). It also decreased the tight junction proteins (TJPs) such as occludin expression, markedly decreasing the RPE cell transepithelial permeability. These studies pinpoint the role of Aβ in RPE alterations and dysfunctions, leading to retinal degeneration. In contrast, the other results revealed that robust expression of the human amyloid‐beta precursor protein (APP) transgene in the retinas of transgenic mice, but a lack of identifiable retinal pathology during the period when Aβ deposits were dramatically escalating in the brain (Chidlow et al., 2017). Although discrete amyloid deposits can be detected in living AD patients (Koronyo et al., 2017), the mechanism underlying the Aβ‐induced retinal pathology remains unclear.

Cell transplantation is a promising experimental therapy for the treatment of retinal degenerative disease. Mesenchymal stem cell (MSC) might protect neuroretina and RPE from further degeneration by replacing the dead and damaged cells as well as secreting many neuroprotective and regenerative factors (McGill et al., 2019). To our knowledge, little information is available about the effects of mesenchymal stem cell‐conditioned medium (MSC‐CM) on the retinal pathology in Aβ‐induced rat model of AD and the underlying mechanisms.

Therefore, we examined the effects of Aβ_1‐42_ oligomer on the retinal pathology *in vivo* after bilateral intracerebroventricular (i.c.v.) injection in rats and human adult retinal pigment epithelial cell line (ARPE‐19) cultures *in vitro*. First, Aβ_1‐42_ was administrated into the bilateral cerebral ventricle of rats to make the AD model *in vivo* (Chang et al., 2016). Next sought to determine whether Aβ application caused ARPE‐19 cell degeneration and apoptosis. Our study shows that MSCs conditioned medium treatment of intraventricular Aβ_1‐42_‐microinjected rats could prevent learning and memory deficits and also reduce retinal pathology, which may via the molecular level of SIRT1/pAKT/GSK‐3β/β‐catenin signaling, TJPs and apoptosis pathways.

## 2 RESULTS

### 2.1 Effect of Aβ‐induced spatial memory, motor, and learning memory impairment for different groups of rats

**FIGURE 1:**
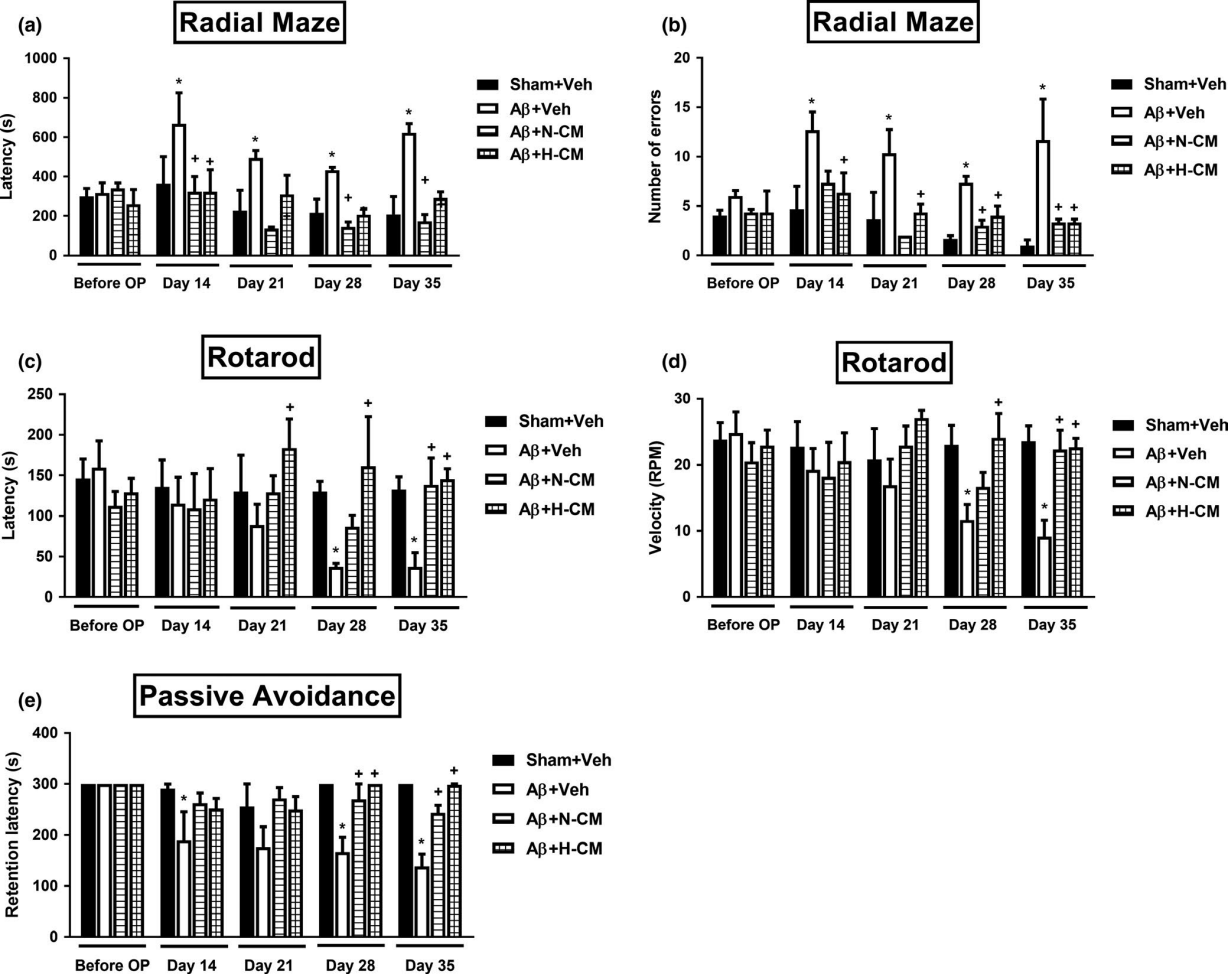
Learning and memory performance and motor activity for different groups of rats. (a) Retention time (Latency period, second) and (b) the number of errors on the radial‐arm maze, the (c) latency (s) and (d) mean velocity (speed, RPM) on rotarod test, and (e) retention latency on passive avoidance were measured in vehicle‐treated sham operation rats (Sham+Veh), vehicle‐treated Aβ injection rats (Aβ+Veh), normoxia mesenchymal stem cell‐conditioned medium treated Aβ injection rats (Aβ+N‐CM), and hypoxia mesenchymal stem cell‐conditioned medium treated Aβ injection rats (Aβ+H‐CM). Data are present mean ± SD. The number of animals used was n = 6 for each experimental group. *p < 0.05 versus the Sham + Veh group; +*p* < 0.05 versus the Aβ+Veh group

As shown in Figure 1a, at day 14 to 35 after Aβ_1‐42_ injection, the vehicle‐treated (Aβ+Veh) group performed poorly cognitive functions as indicated by a longer latency period when compared to the non‐Aβ control (or sham+Veh group), which ultimately indicates that Aβ_1‐42_ disrupts the long‐term spatial memory. The significant change in the latency period was observed in both rats treated with the conditioned medium under normoxia (N‐CM) or hypoxia (H‐CM) groups followed Aβ administration, as shown in Figure 1a. The working memory errors were also significantly increased in the Aβ+Veh group compared with the Sham+Veh group (Figure 1b). Motor performance and balance skills of rats received Aβ_1‐42_ were evaluated on an accelerating rotating rod. Figure 1c,d showed the mean of time (latency) and velocity that rat on the rod. The Aβ+Veh rats significantly decreased both the latency (Figure 1c) and velocity (Figure 1d) on the rotarod at day 28 to day 35 after Aβ injection. Both the Aβ+N‐CM and Aβ+H‐CM group of rats demonstrated improvement in rotarod activity as compared to Aβ+Veh group, which provided significant functional recovery of locomotor activity. As shown in Figure 1e, the retention latency of a passive avoidance task in Aβ+Veh rats was significantly shorter than that of Sham+Veh rats. Again, the learning and memory deficits caused by Aβ_1‐42_ were significantly attenuated by both N‐CM and H‐CM (Figure 1e).

### 2.2 N‐CM and H‐CM attenuates the Aβ‐induced histochemical alterations of NFL, GCL, photoreceptor, and RPE in rats

**FIGURE 2:**
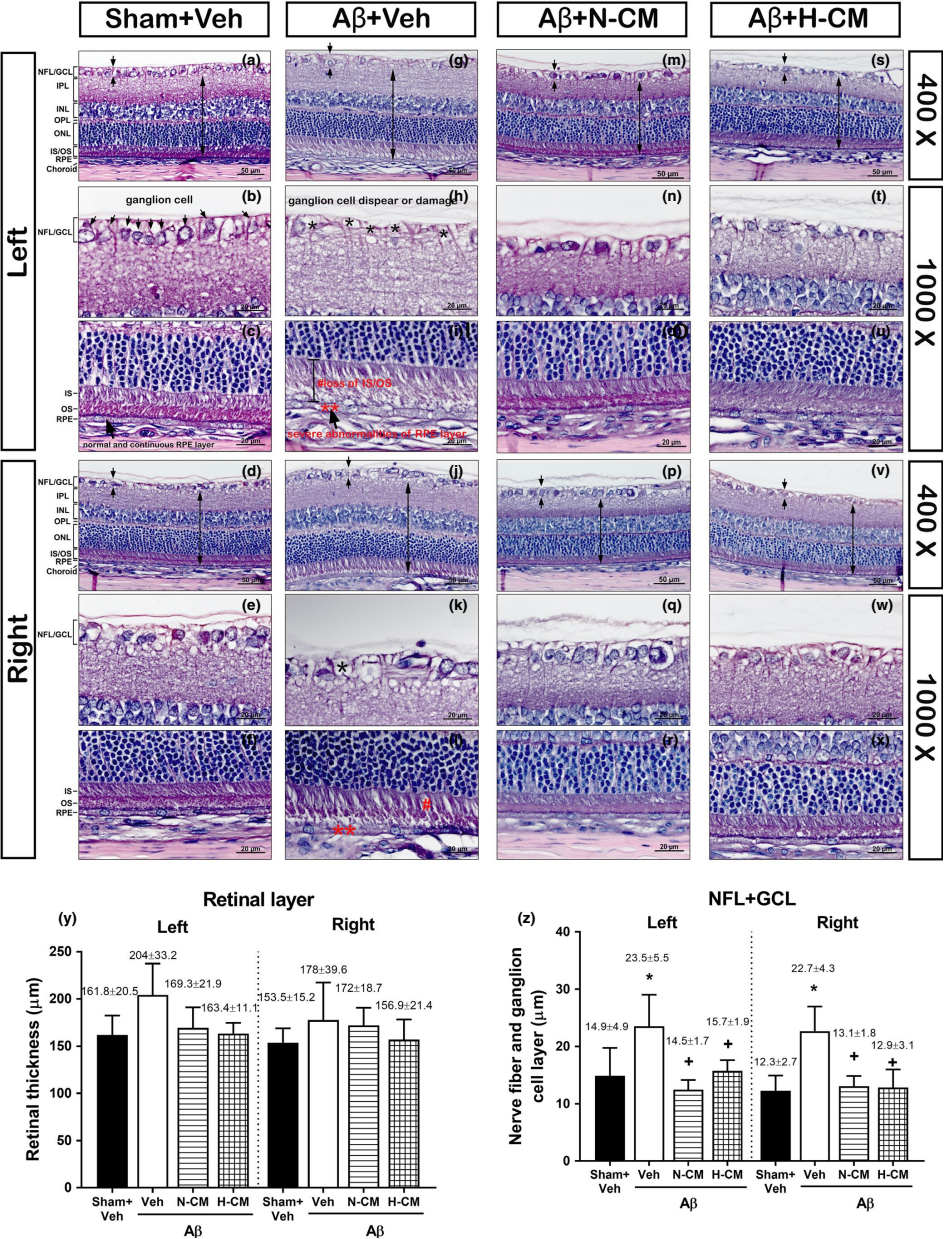
Histology of the rat retina (left or right side) using H & E staining. The micrograph shows representative left and right eye from Sham+Veh group (a‐f), Aβ+Veh (g‐l), Aβ+N‐CM (m‐r), and Aβ+H‐CM (s‐x). The black arrow indicates retinal thickness or NFL plus GCL thickness. Values for retinal thickness (y) and nerve fiber and ganglion cell layer (z) are shown. Values are shown as means ± SD (n = 6 of each group). *p < 0.05, compared with the Sham+Veh group; +*p* < 0.05, compared with the Aβ+Veh group. NFL, nerve fiber layer; GCL, ganglion cell layer; IPL, inner plexiform layer; INL, inner nuclear layer; OPL, outer plexiform layer; ONL, outer nuclear layer; IS, inner segments; OS, outer segments; RPE, retinal pigment epithelium. Black star (*) indicates the loss of ganglion cells; red star (**) indicates the loss of RPE cells; and hashtag (#) indicates the loss of IS/OS. Scale bar = 20 μm and 50 μm

Figure 2 depicted the effect of bilateral intracerebroventricular (i.c.v.) injection of Aβ_1‐42_ on the retinal morphology of both eyes in rats. H & E staining revealed that Sham+Veh rats displayed regular and clear layers and no significant morphological change in right and left retina (Figure 2a–f). However, five weeks after Aβ_1‐42_ injection, the Aβ+Veh rats showed disruption and hypertrophy of the nerve fiber and ganglion cell layer (NFL+GCL) in both right and left retina (Figure 2g–l). Examination at a higher level of magnification (100 X objective lens) also confirmed the loss of integrity of GCL (Figure 2h,k), hypopigmented and disorganized RPE cells, as well as an alteration of the photoreceptors with loss of inner and outer segments (IS/OS) (Figure 2i,l) after Aβ injection. Hypertrophy or disappearance of RPE was another marked alterations observed after the Aβ injection (Figure 2i,l). Compared to the Sham+Veh group rats, the Aβ+Veh rats had a significant lower retinal thickness and higher thickness of the NFL+GCL layer in both eyes (Figure 2y,z). Compared with the Aβ+Veh group, the N‐CM and H‐CM treated groups were also seen as the loss or shorting of IS and OS, but preserved the RPE (Figure 2m–x). The thickness of inner retina including inner plexiform layer (IPL), inner nuclear layer (INL), outer plexiform layer (OPL), outer nuclear layer (ONL), and IS/OS trends to decrease at 5 weeks after Aβ exposure without statistical significance (Figure 2y). The thickness of the NFL+GCL was significantly increased at week 5 after exposure to Aβ compared with the control (Sham+Veh) group, showing moderate swelling (Figure 2z). The Aβ‐induced histological alternations of NFL, GCL, and RPE were all significantly reduced by N‐CM or H‐CM (Figure 2n,o,q,r,t,u,w,x). Further, N‐CM‐ and H‐CM‐treated rats showed a minor reduction of the photoreceptor layer, maintained GCL and RPE integrity (Figure 2n,o,q,r,t,u,w,x), and thinner nerve fiber and ganglion cell layers than did Aβ+Veh rats (Figure 2y).

### 2.3 N‐CM or H‐CM inhibits the Aβ‐induced degeneration and apoptosis of both retinal ganglion cells (RGC) and retinal pigment epithelial (RPE) cells in rats

**FIGURE 3:**
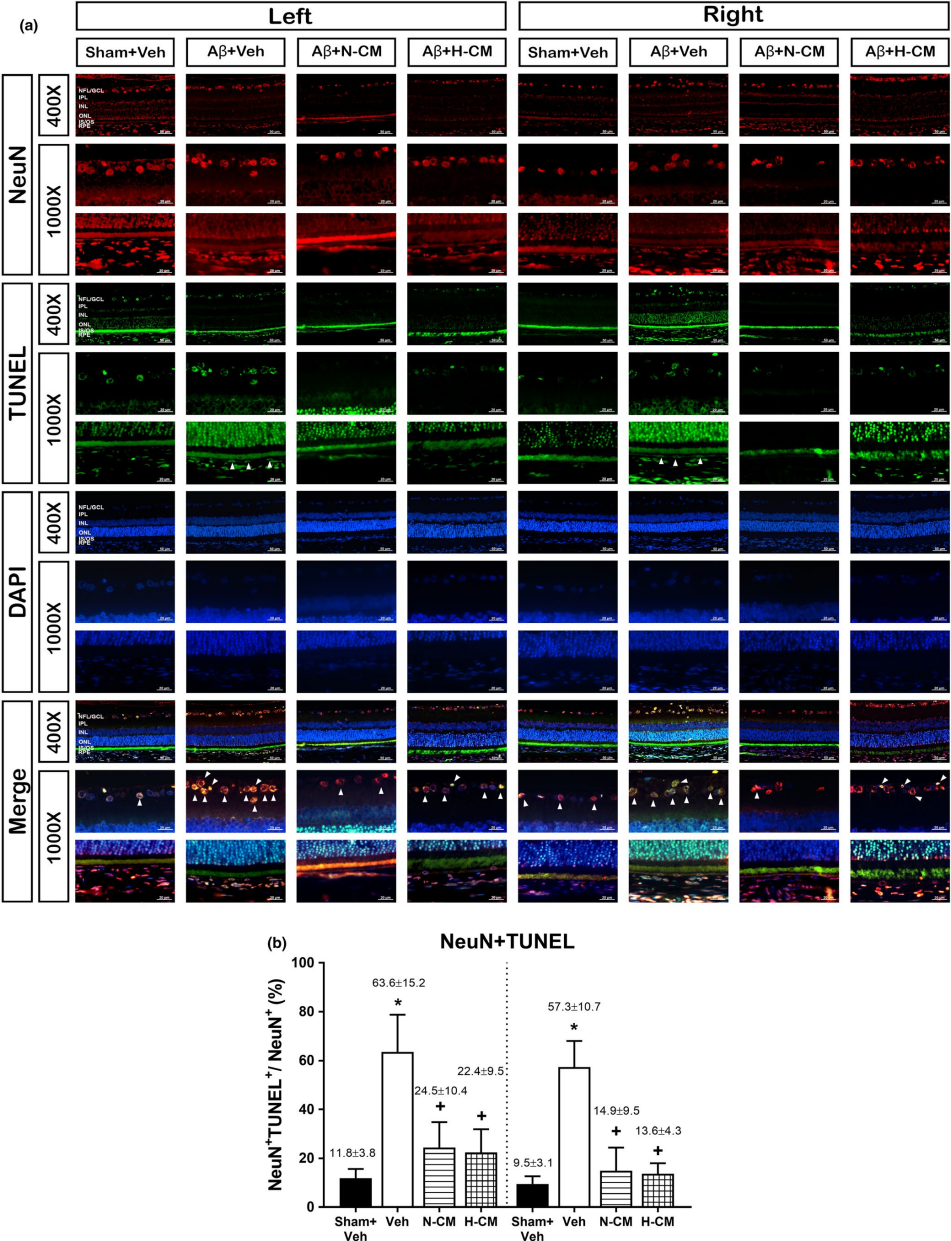
TUNEL staining of rat retina (left and right side) for different groups of rats. (a) Representative images (400X and 1000X) of apoptotic cells were marked with green fluorescence (TUNEL), the nuclei of cells are stained by blue fluorescence (DAPI), and retina ganglion cells are stained by red fluorescence (NeuN). Arrowhead sign indicates apoptotic neuron signal. (b) The number of TUNEL‐positive cells in the GCL was significantly greater in the Aβ‐Veh retina than those of the Sham+Veh retina. Data are expressed as means ± SD (N = 6 for each group). *p < 0.05 compared with the Sham+Veh group. +*p* < 0.05 compared with the Aβ+Veh group. Scale bar = 20μm and 50 μm

We performed Fluoro‐Jade B (FJB, a marker of a degenerative cell) stain (Supplementary Figure S2a) and TUNEL (a marker of an apoptotic cell) assay (Figure 3a and Supplementary Figure S3) to quantify the number of degenerative and apoptotic cells in both the GCL and RPE in rats, respectively. FJB staining showed that, compared to the Sham+Veh group rats, the Aβ+Veh group rats had significantly higher % degenerative neurons in both the left and right eye (Supplementary Figure S2b). Immunofluorescence staining also showed that compared to the Sham+Veh group rats, the Aβ+Veh group rats had significantly higher % of apoptotic cells in the GCL and RPE in both the left and right eyes at week 5 after Aβ injection (Figure 3b). After co‐labeling with FJB staining or TUNEL staining and NeuN, both of degenerative and apoptotic neurons in the GCL were found to be mostly RGCs. The increased % of both degenerating neurons and apoptotic neurons in both left eye and right eye following i.c.v. injections of Aβ were significantly reduced by N‐CM or H‐CM.

### 2.4 N‐CM or H‐CM attenuate Aβ‐induced β‐catenin downregulation in RGC and RPE cells in rats

Immunofluorescence staining was performed to evaluate β‐catenin expression in response to retinal stress caused by Aβ. The data revealed that β‐catenin expression in all layers of the retina in the Sham+Veh group (Supplementary Figure S4). The β‐catenin and NeuN co‐labeling were evident in the RGC of GCL. Aβ‐injection in experimental rats, this expression significantly reduced in Aβ+Veh rats. At week five, after N‐CM or H‐CM administration, β‐catenin was densely expressed in the GCL and RPE layer in Aβ injection rats.

### 2.5 N‐CM or H‐CM maintain the tight junction protein expression in ARPE‐19 cells following Aβ administration

**FIGURE 4:**
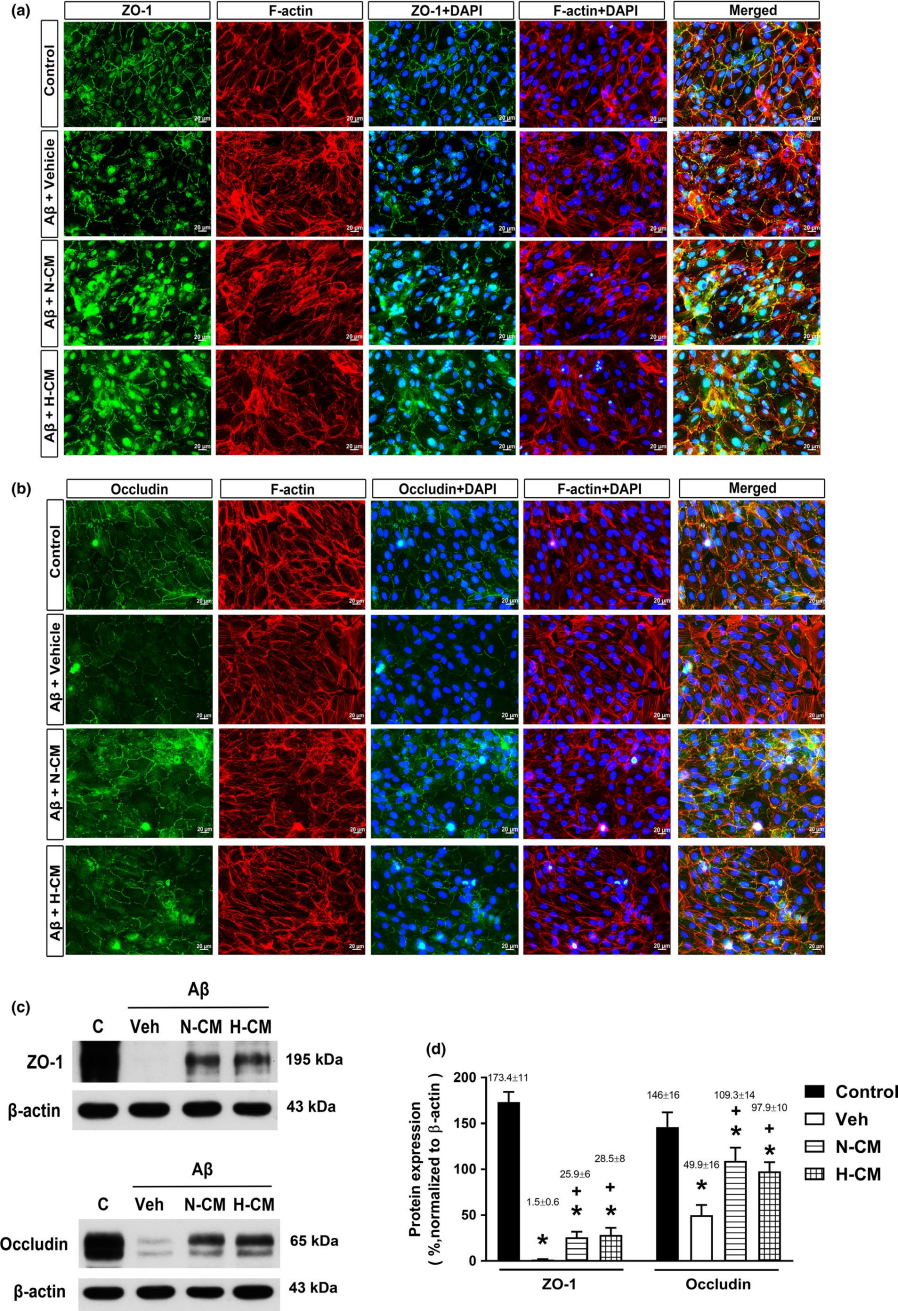
Evaluation of tight junction protein (ZO‐1 and occludin) and cytoskeletal microfilaments (F‐actin) of ARPE‐19 cells treated with or without Aβ. Immunofluorescence expression of (a) ZO‐1 and (b) occludin. Scale bar= 20 μm. (c) Western blot analysis of ZO‐1 and occludin, and β‐actin was used as a loading control. (d) Quantification of densitometric scans of protein bands showing a significant decrease in ZO‐1 and occludin expression in each group. Values are the means ± SD of three independent experiments. *p < 0.05 compared with Control; +*p* < 0.05 compared with Aβ+Veh group

The alteration we observed in the retinal structure disorganization of Aβ‐treated rats led us to study the effects of N‐CM and H‐CM on the ARPE‐19 cell barrier function. As shown in Figure 4a,b, continuous ZO‐1 and occludin were observed between ARPE‐19 cells in the control group, while attenuated, interrupted, and diminished ZO‐1 and occludin were observed between ARPE‐19 cells treated with Aβ for 24 h. These tight junction proteins (TJPs) were confirmed with Western blot analysis (Figure 4c,d), showing that Aβ decreased TJPs expression. In addition, we examined the expression of F‐actin in ARPE‐19 under Aβ stimulation. We observed the normal and continuous expression of F‐actin in the control group, while interrupted F‐actin in the Aβ+Veh group (Figure 4a,b). The areas where F‐actin was disrupted also showed interrupted ZO‐1 and occludin expression. These data presumably reflect a tight junction and cytoskeleton disorganization in Aβ‐mediated ARPE‐19 cells. The interrupted ZO‐1 and occludin expression caused by Aβ in ARPE‐19 cells was significantly reduced by treatment with N‐CM or H‐CM (Figure 4).

### 2.6 N‐CM or H‐CM alleviate Aβ‐induced cell morphology alterations and decreased viability in ARPE‐19 cell

**FIGURE 5:**
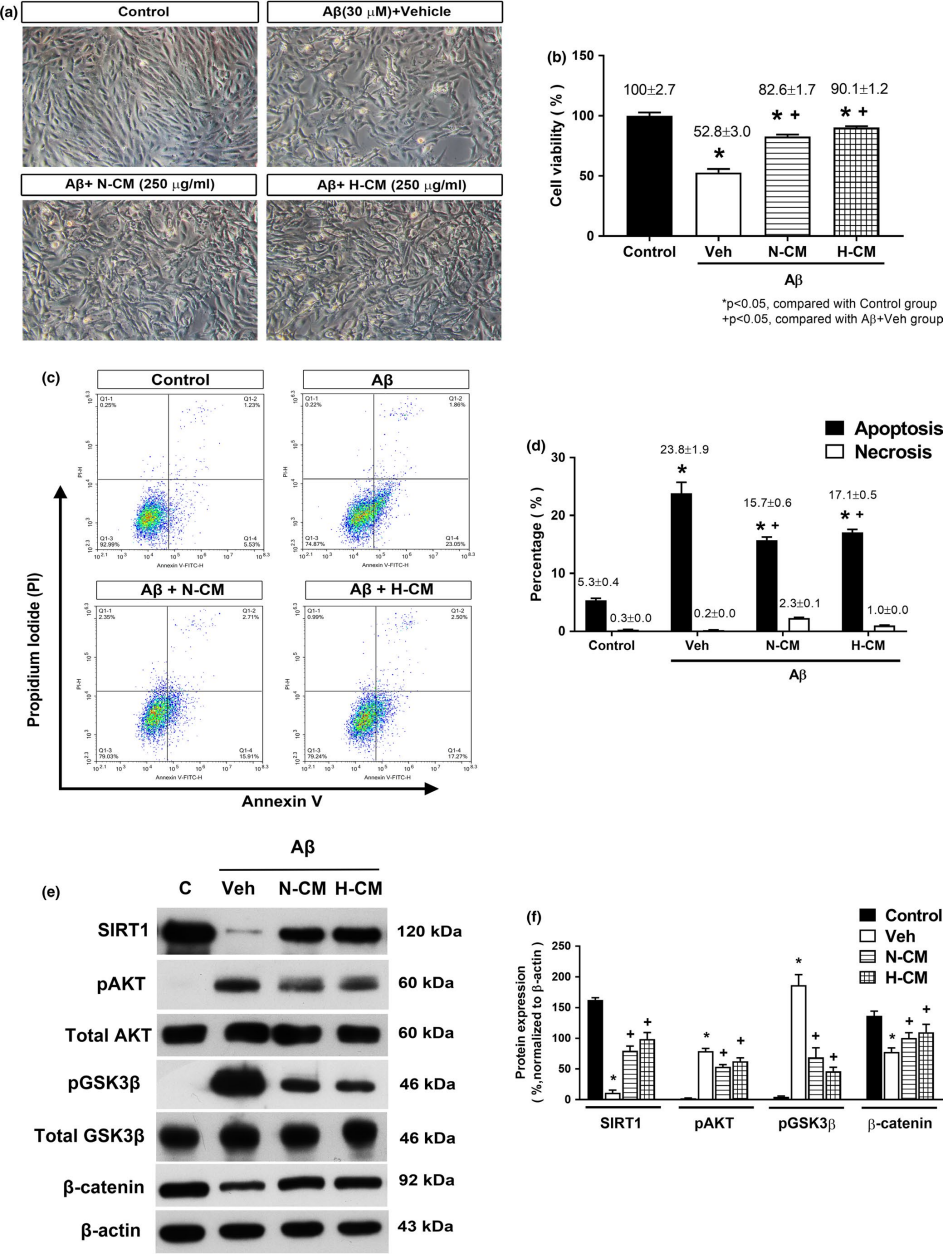
Cell viability and apoptotic rate of the ARPE‐19 cells treated with 30 μM Aβ. ARPE‐19 cells were treated with 250 μg/ml N‐CM or 250 μg/ml H‐CM for 24 h after Aβ treatment. (a) Representative photographs of ARPE‐19 morphological changes under inverted phase‐contrast microscopy (x200) from non‐treated cells (Control group); cells treated with 30 μM Aβ for 24 h, and then cultured for another 24 h after addition of DMEM (as a vehicle) (Aβ+Veh group); cells treated with 30 μM Aβ for 24 h, and then cultured for another 24 h after addition of 250 μg/ml N‐CM (Aβ+N‐CM group); and cells treated with 30 μM Aβ for 24 h, and then cultured for another 24 h after addition of 250 μg/ml H‐CM (Aβ+H‐CM group). (b) MTT assay was performed after treatment with N‐CM and H‐CM for 24 h, following pre‐incubation with Aβ for 24 h. Relative cell viability was calculated from the optical density value at 472 nm against that of the Control group. (c) Analysis of early and late apoptosis detected by Annexin V/PI double staining. (d) Data of three sets of independent experiments were quantified. (e) Western blot analysis of SIRT1, phosphor (p)‐ and total AKT, phosphor (p)‐ and total GSK3β, and β‐catenin proteins in ARPE‐19 cells treated with or without Aβ. β‐actin served as the loading control. (f) The graph depicts the densitometric analysis of the bands for each group. *p < 0.05 compared with the Control group; +*p* < 0.05 compared with the Aβ+Veh group. *p < 0.05 compared with Control; +*p* < 0.05 compared with the Aβ+Veh group

Phase‐contrast micrographs of ARPE‐19 cells following treatment with Aβ showed that the cell morphology became irregular and shrunken, with increased dead cells (Figure 5a). The number of shrunken and apoptotic cells caused by Aβ induction was decreased following treatment with N‐CM and H‐CM. MTT assay showed that 30 μM Aβ stimulation caused an approximate 50% reduction in RPE cell viability (Figure 5b). Post‐treatment with N‐CM or H‐CM at a concentration of 250 μg/ml significantly inhibited Aβ‐induced reduction of cell viability.

### 2.7 N‐CM and H‐CM inhibit Aβ‐induced apoptosis in ARPE‐19 cells

The above results have shown that N‐CM and H‐CM inhibited Aβ‐induced ARPE‐19 cell death. Next, we examined whether the protective effect of N‐CM and H‐CM was due to apoptosis prevention. The annexin V/ PI flow cytometry analysis was used to test the apoptosis of ARPE‐19 cells. Data are expressed as % of Annexin V‐FITC‐positive and PI‐negative cells (early stage of apoptosis). As shown in Figure 5c,d, the percentage of apoptotic cells in the Aβ+Veh group was significantly higher than that in the control group (23.8 ± 1.9% vs. 5.3 ± 0.4%). Compared to the Aβ+Veh group, the N‐CM or H‐CM group had a significantly decreased percentage of apoptosis cells (N‐CM: 15.7 ± 0.6% vs. 23.8 ± 1.9%; H‐CM: 17.1 ± 0.5% vs. 23.8 ± 1.9%).

### 2.8 N‐CM or H‐CM inhibit the Aβ‐induced ARPE‐19 injury by activating SIRT/β‐catenin signaling pathway

**FIGURE 6:**
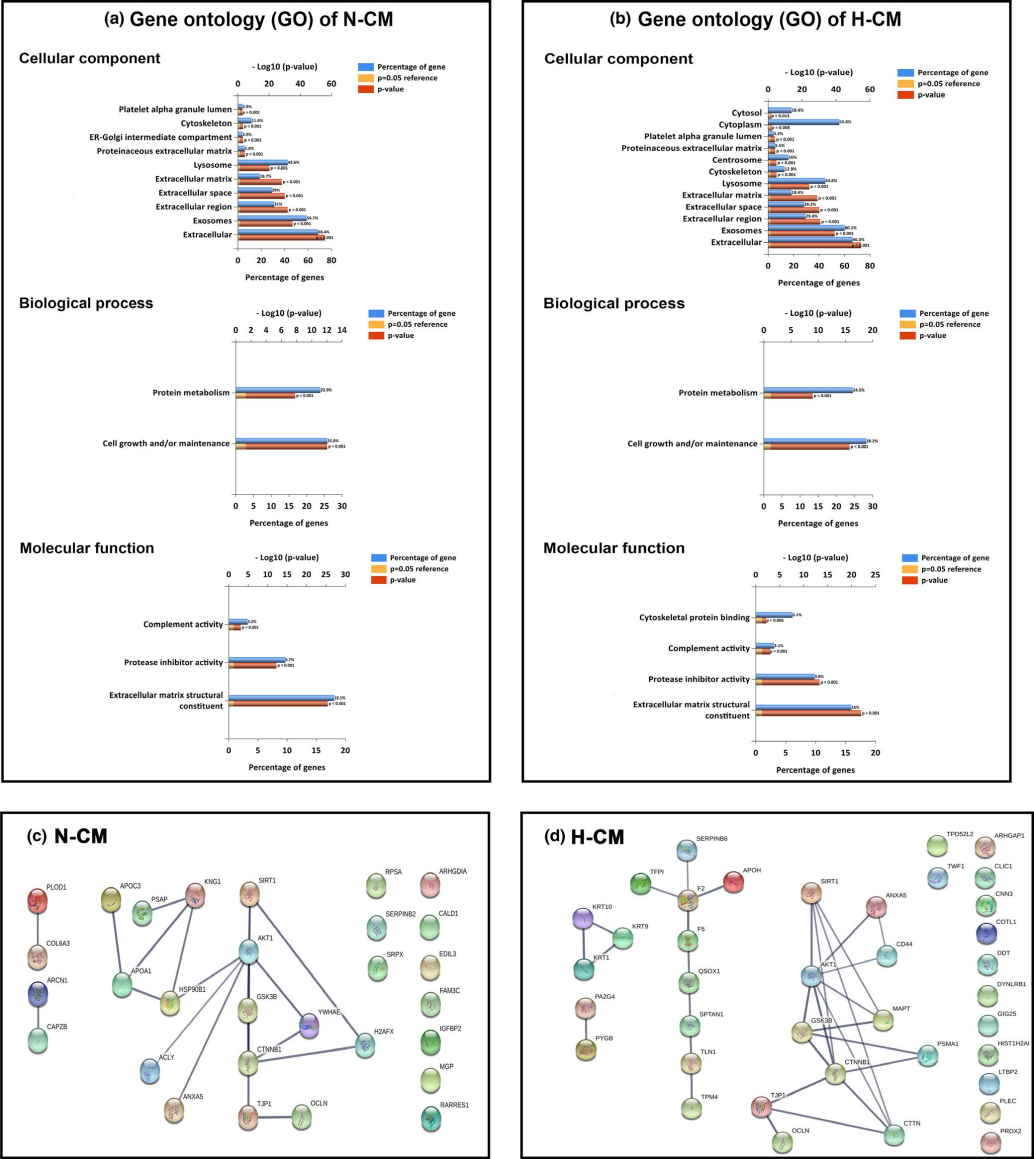
Gene Ontology (GO) and subcellular location analysis of the differentially expressed peptides obtained from N‐CM (a,c) and H‐CM (b,d). Cellular process, biological regulation, and cellular component organization were the most highly enriched biological processes. The 15 in N‐CM (a) and 18 in H‐CM (b) significant GO terms were listed for cellular component, biological process, and molecular function. The blue column shows the percentage of genes, while the red column shows the p‐value (–log10). STRING representation of the interactions of the 22 and 30 proteins found in N‐CM (c) and H‐CM (d) samples, respectively. The analysis was set for high confidence with the required confidence (score) 0.700. The thicker the black line, the stronger the connection between these proteins. Connections are based on seven criteria: co‐mentioned in PubMed articles (text mining), experiments, databases, co‐expression, neighborhood in the genome, gene fusion, and co‐occurrence across genomes. We also added the SIRT1, AKT1, GSK3B, CTNNB1 (β‐catenin), TJP1 (tight junction protein), OCLN (occludin gene), ANXAS (annexin V), and MAPT (tau) to predict the relationship between SIRT1/pAKT/pGSK3β/β‐catenin, tight junction, apoptosis pathway and the unique conditioned medium components of mesenchymal stem cells

Results from Figure 5e,f demonstrated that Aβ+Veh group rats had significantly lower levels of both sirtuin 1 (SIRT1) and β‐catenin than did the Sham+Veh group rats. In contrast, compared to the Sham+Veh group rats, the Aβ+Veh group rats had significantly higher phosphorylation levels of both pAKT and pGSK3β (Figure 5e,f). Both the downgrade levels of SIRT1and β‐catenin and the upgrade levels of pAKT and pGSK3β in the ARPE‐19 treated with Aβ were all significantly reduced by N‐CM or H‐CM (Figure 5e,f). The MS/MS analysis revealed that N‐CM contains 22 unique proteins out of 155 proteins (Supplementary Table S1), whereas H‐CM contains 30 unique proteins out of 163 proteins (Supplementary Table S2). Next, cellular components, biological processes, and molecular functions of the corresponding proteins were determined by GO analysis to predict the latent functions of the differentially expressed peptide (Figure 6a,b). STRING found 8 proteins out of the 22 proteins (components of N‐CM) in Supplementary Table S1 interacted with SIRT1/ pAKT/ pGSK3β/ β‐catenin signaling, TJPs, and apoptosis pathways (*p* < 0.05). Interaction network analysis was performed using STRING (search tool for the retrieval of interacting genes/proteins, http://string‐db.org/, version 11.0). The STRING interaction network model is shown in Figure 6c, with the known functions of the proteins in Supplementary Table S1. In addition, STRING found 3 proteins out of 30 proteins in Supplementary Table S2 (components of H‐CM) interacted with SIRT1/ pAKT/ pGSK3β/ β‐catenin signaling, TJPs, and apoptosis pathways (*p* < 0.05). The STRING interaction network model is shown in Figure 6d, with the known functions of the proteins in Supplementary Table S2.

## 3 DISCUSSION

Many AD patients have visual impairments (Ramzaoui et al., 2018). Compared to age‐matched controls, AD patients have RGC loss, retinal nerve fiber layer thinning, and axonal degeneration (Chan et al., 2019). Meanwhile, amyloid plaques or Aβ accumulations have been detected in retinas of AD patients or transgenic mouse models of AD in an advanced stage of the disease (Dewing et al., 2019; Singh & Verma, 2020). The aim of the current study was to characterize retinal pathology *in vivo* after a single intracerebroventricular injection of Aβ_1‐42_ in rats and *in vitro* after the rapid application of Aβ_1‐42_ on ARPE‐19 cells. First, 14–35 days after bilateral i.c.v. Aβ_1‐42_ injection, animals displayed both motor and cognitive deficits and retinal pathology. Our *in vivo* results revealed that Aβ_1‐42_ injection caused disruption and hypertrophy of both nerve fiber layer and ganglion cell layer, thinning of the photoreceptor layer with loss of inner and outer segments, hypertrophy and disappearance of retinal pigment epithelium (RPE), and degeneration and apoptosis of both retinal ganglion cells (RGC) and RPE cells in rats up to 35 days post‐injection. Our study pinpoints the role of Aβ_1‐42_ in retinal pathology in AD‐like rats and suggests targeting Aβ_1‐42_ may help develop selective methods for treating disease involving retinal pathology. Second, *in vitro* studies showed that rapid application of Aβ_1‐42_ caused decreased viability, increased apoptosis, and decreased expression of tight junction proteins (occludin and ZO‐1) in ARPE‐19 cells. Structures of occludin and ZO‐1 are crucial to the development and maintenance of RPE morphology and function. It has been demonstrated that SIRT1 activator resveratrol could attenuate Aβ‐induced RPE barrier disruption (Balaiya et al., 2017; Cao et al., 2013) and SRT2172 (a novel SIRT1 small molecule activator) could blocked the increase of MMP‐9 expression which has been reported to modify barrier function by disrupting TJP (Nakamaru et al., 2009). SIRT1 was detected in the cornea, lens, ciliary body, RPE, and neuroretina in rodents and humans, and in the human normal conjunctival epithelium (Zhou et al., 2018). Chen et al. investigated three variants of the SIRT1 gene associated with AMD in Chinese Han individuals (Chen et al., 2015). Kubota et al. have demonstrated that retinal SIRT1 activity reduced significantly in a light‐induced retina injury model (Kubota et al., 2010). In an *in vitro* study, ultraviolet B activated the phosphoinositide‐3 kinase (PI3 K)/ protein kinase B (AKT)/extracellular signal‐regulated kinase (ERK) pathway by reducing the expression of SIRT1 in ARPE‐19 cells and induced cell injury (Chou et al., 2013). Glycogen synthase kinase 3β (GSK3β) has been shown to phosphorylate Tau in intact cells which is involved in the AD pathogenesis (Kitagishi et al., 2014a). GSK3β is ubiquitously active and is a critical effector of PI3 K/AKT cellular signaling which involved in the cellular process such as cell metabolism, cell death, and tauopathy for AD (Kitagishi et al., 2014a). In epithelial cells, the SIRT1 deacetylation of β‐catenin causes the release of β‐catenin from the nucleus (Firestein et al., 2008).

In the present results, exposure to Aβ caused a decrease cytoplasmic distribution of ZO‐1 and occludin of ARPE19 cell and treated with N‐CM or H‐CM inhibited the deleterious effects of Aβ on RPE integrity via maintained ZO‐1, occludin, and SIRT1 expression. These results suggested that N‐CM and H‐CM with its maintained SIRT1 expression properties might reverse the deleterious effects of Aβ on RPE barrier structure.

However, other studies have failed to find differences in RGC density (Curcio & Drucker, 1993) and myelinated axon number (Davies et al., 1995) between AD patients and matched controls. Aβ deposits similar to those in the brain are not identified in the eyes of AD patients (Ho et al., 2014). A well‐validated mouse model of AD revealed that robust expression of the human amyloid precursor protein (APP) transgene in the retina of transgenic mice, but a lack of identified retinal pathology during the period when Aβ deposits were dramatically escalating in the brain (Chidlow et al., 2017). Although intravitreal Aβ caused retinal pathology up to day 14 post‐injection, however, on day 30 post‐injection, the retinal morphology showed a trend toward normalization in rats (Mohd Lazaldin et al., 2018). In our present study, intracerebroventricular injection of Aβ caused GCL hypertrophy. It is interesting to know the melanopsin retinal ganglion cells are affected after Aβ_1‐42_ injection. Examination of postmortem AD retinal specimens revealed that age‐related loss of optic nerve axons and specifically melanopsin RGC pathology were associated with Aβ deposition (La Morgia et al., 2016).

Previous studies showed that intravitreal injection of bone marrow MSC‐CM 24 h after retinal ischemia significantly improved retinal function and attenuated cell loss in the RGC layer of adult Wistar rats (Dreixler et al., 2014). By spectral counting, compared to unconditioned medium, 19 proteins that met stringent identification criteria were in the conditioned medium obtained from bone marrow stem cells. The majority of those proteins were involved in cell growth and adhesion in an interactional network.

Previous studies have shown that intravitreal delivery of neurotrophic factors slow down photoreceptor degeneration in rodent glaucoma and optic tract trauma model, but the effect was temporary (Abulfadl et al., 2018). In our present study, slow‐release neurotrophins by implantation of ALZET osmotic minipumps containing N‐CM or H‐CM from MSC on day 7 after an i.c.v. injection of Aβ significantly protected against the motor and cognitive deficits as well as the retinal pathology in rats. In addition, the application of N‐CM or‐CM significantly improved the Aβ_1‐42_‐induced decreased viability, increased apoptosis, decreased expression of tight junction proteins, increased expression of both pAKT and pGSK3β and decreased expression of both SIRT1 and β‐catenin in ARPE‐19 cell culture. The mass spectrometry analysis revealed that N‐CM contains 22 unique proteins, whereas H‐CM contains 30 unique proteins (Supplementary Table S1 and Supplementary Table S2). GO analysis and STRING analysis found that 8 proteins out of 22 proteins form N‐CM and 3 proteins out of 30 proteins from H‐CM interacted with SIRT1/pAKT/pGSK3β/β‐catenin signaling, tight junction proteins, and apoptosis pathways. Bioinformatics analysis showed that these differentially expressed peptides might be associated with the beneficial effects exerted by MSC‐CM in treating retinal pathology.

Our present results are consistent with many previous experiments. For example, the key to successful retina regeneration is Müller glia (MG), the primary glial cell type in the retina (Bernardos et al., 2007). In mammals, β‐catenin has been associated with MG proliferation (Osakada et al., 2007; Osakada & Takahashi, 2009). Nuclear factor‐kappa B (NF‐κB), a key regulator of the inflammatory response is modulated by reversible acetylation of the NF‐κB RelA/p65 subunit (Chen et al., 2001). Full transcription activity of RelA/p65 requires acetylation of Lys310, which can be deacetylated by sirtuin (SIRT1) (Yeung et al., 2004). An activation of SIRT1 (e.g., Resveratol) inhibits NF‐κB signaling by promoting the deacetylation of Lys310 of RelA/p65 (Chen et al., 2001). Thus, SIRT1 can be a key negative regulator of inflammation cells via inhibition of NF‐κB activation. In our present study, Aβ might induce pro‐inflammatory cytokine production and blood–retinal barrier disruption in human adult retina pigment epithelium cells by inhibiting SIRT1. Retinal injury, growth factors, and cytokines converge on β‐catenin and pStat3 signaling to stimulate retina regeneration (Wan et al., 2014). The phosphoinositide 3‐kinase (PI3K)/AKT/GSK3β pathway has been shown to play a pivotal role in neuroprotection, enhancing cell survival by stimulating cell proliferation and inhibiting apoptosis. This pathway promotes protein hyperphosphorylation in Tau protein (Kitagishi et al., 2014b; Morroni et al., 2016), which is one of AD pathological features. Donepezil a therapeutic acetylcholinesterase inhibitor being used for the treatment of AD. It has been proposed that donepezil prevents glutamate neurotoxicity through the PI3K/AKT/GSK3β signaling (Haraguchi et al., 2017; Kitagishi et al., 2014b).

This study investigated the *in vivo* and *in vitro* effects of the Aβ_1‐42_ on retinal pathology and found that it caused disruption and hypertrophic of the nerve fiber layer and ganglion cell layer, reduced thickness of photoreceptor layer, hypertrophic and disorganization of retinal pigment epithelium, and degeneration and apoptosis of retinal ganglion cells and retinal epithelial pigment cells in rats 35 days following i.c.v. injection of Aβ_1‐42_. Additionally, *in vitro* studies showed that ARPE‐19 cells following Aβ_1‐42_ application had decreased viability along with apoptosis and decreased expression of tight junction proteins. Western blot analysis revealed that Aβ_1‐42_ caused increased expression of both pAKT and pGSK3β and decreased expression both of SIRT1 and β‐catenin in ARPE‐19 cells. Conditioned medium derived from mesenchymal stem cells under normoxia or hypoxia conditions protected against Aβ_1‐42_‐induced retinal pathology in both rats and ARPE‐19 cells culture. We used mass spectrometry to compare the peptides profile of MSC‐CMs. Gene ontology enrichment analysis and STRING analysis were conducted to explore the differentially expressed peptides by predicting the functions of their precursor proteins. Bioinformatics analysis showed that 8 out of 155 proteins in the N‐CM and 3 out of 163 proteins in the H‐CM might be associated with SIRT1/pAKT/pGSK3β/β‐catenin, tight junction proteins, and apoptosis pathways. Thus, the secretome information on MSC‐CMs may be helpful for the prevention and treatment of retinal pathology in many neurodegenerative diseases.

## 4 EXPERIMENTAL PROCEDURES

All authors had access to the study data and reviewed and approved the final manuscript. Materials and methods are described in detail in the supplementary section. All *in vivo* studies represent 20 animals per group. All *in vitro* studies are from at least 3 replicate experiments. All animal experiments were approved and carried out in accordance with the Institutional Animal Care and Use Committee at the Chi Mei Medical Center (Tainan, Taiwan). Data are presented as the mean ± standard deviation. Schematic diagrams showing the *in vivo* and *in vitro* experimental designs are shown in Supplementary Figure S1.

## CONFLICT OF INTEREST

The authors declare that they have no competing interests.

## AUTHOR CONTRIBUTIONS

SCK and CCC involved in study concept and design. CHY, JTM, and WPL involved in acquisition of data. SCK and CHY involved in analysis and interpretation of data. CCC, CPC, and KCL involved in drafting of the manuscript. SCK and CCC involved in critical revision of the manuscript for important intellectual content. JTM and WPL involved in statistical analysis. CCC and CPC involved in material support. CCC, CPC, and KCL involved in obtained funding.

## Supporting information

Supplementary Material

Fig S1

Fig S2

Fig S3

Fig S4

## Data Availability

The authors confirm that the data supporting the findings of this study are available within the article and supplementary materials.
