# Supplementary material for: Mesenchymal stem cell‐conditioned medium attenuates the retinal pathology in amyloid‐β‐induced rat model of Alzheimer's disease: Underlying mechanisms
Source: Aging Cell. 2021 Mar 30;20(5):e13340. doi: 10.1111/acel.13340 (PMC8135003; doi:10.1111/acel.13340)
Supplement: Supplementary file 1 — Supplementary Material [file ACEL-20-e13340-s003.docx]

**Supplementary File**

**Mesenchymal stem cell-conditioned medium attenuates the retinal pathology in amyloid-β-induced rat model of Alzheimer's disease: underlying mechanisms**

**Shu-Chun Kuo^1,2^, Chung-Ching Chio^3^, Chao-Hung Yeh^2,3^, Jui-Ti Ma^4^, Wen-Pin Liu^4^, Mao-Tsun Lin^4^, Kao-Chang Lin^5,6,*^, Ching-Ping Chang^4,*^**

^1^Department of Ophthalmology, Chi Mei Medical Center, Tainan, Taiwan

^2^Department of Optometry, Chung Hwa University of Medical Technology, Tainan, Taiwan

^3^ Division of Neurosurgery, Department of Surgery, Chi Mei Medical Center, Tainan, Taiwan

^4^Department of Medical Research, Chi Mei Medical Center, Tainan, Taiwan

^5^Department of Holistic Care, Chi Mei Medical Center, Tainan, Taiwan

^6^Department of Neurology, Chi Mei Medical Center, Tainan, Taiwan

**Correspondence**

**Kao-Chang Lin**, MD.

Department of Holistic Care, Chi Mei Medical Center, Tainan, Taiwan

Add: No. 901, Zhonghua Rd., Yongkang District, Tainan City 710, Taiwan

Email: [gaujang@mail2000.com.tw](mailto:gaujang@mail2000.com.tw)

**Ching-Ping Chang**, Ph.D. Department of Medical Research, Chi Mei Medical Center, Tainan, Taiwan.

Add: No. 901, Zhonghua Rd., Yongkang District, Tainan City 710, Taiwan

Email: [jessica.cpchang@gmail.com](mailto:jessica.cpchang@gmail.com) or [a50831@mail.chimei.org.tw](mailto:a50831@mail.chimei.org.tw)

**Experimental Procedures**

**1. Animal**

We purchased male Wistar rats (weight of 250–350 g) from BioLASCO Co., Ltd Taiwan (Taipei, Taiwan) and housed in an air-conditioned animal facility at 26 ± 0.5°C under a 12-hour light-dark cycle and water and food provided *ad libitum* at least two weeks before the start of experimentation. All behavioral tests were conducted between 10:00 a.m. and 04:00 p.m. The study protocols were approved by the Institute Animal Care and Use Committee of Chi Mei Medical Center (IACUC approved no. 107121209). We used the ARRIVE checklist when writing our report.

**2. Experimental groups and surgical procedures**

Eighty Wistar rats were randomly assigned into the following four groups: (1) Sham operation control group treated with vehicle solution (Sham+Veh, n=20), (2) Aβ_1-42_-treated group received vehicle solution( Aβ+Veh, n=20), (3) Aβ_1-42_-treated group received normoxia MSC conditioned medium (Aβ+N-CM, n=20), and (4) Aβ_1-42_-treated group received hypoxia MSC conditioned medium (Aβ+H-CM, n=20). The low glucose DMEM was used as a vehicle solution.

Rats were anesthetized with a mixture of Zoletil (40 mg/kg; Virbac, Nice, France), xylazine hydrochloride (2 mg/kg; Balanzine, Health-Tech Pharmaceutical Co., Taipei, Taiwan), and atropine sulfate (1 mg/kg; Tai Yu Chemical & Pharmaceutical Co. Ltd., Hsinchu, Taiwan) and then placed in a Kopf stereotaxic apparatus (David Kopf Instruments, Tujunga, CA, USA). The rectal temperature of each rat was kept at 37°C±0.5°C using a Homeothermic control unit (RightTemp® Temperature Monitor & Homeothermic Warming Control Module; Kent Scientific, Torrington, CT, USA). A middle sagittal incision was made in the scalp using standard sterilized procedures. Bilateral holes were drilled in the skull using a dental drill over the lateral ventricles (LV). Bilateral intracerebroventricular (ICV, AP: -0.8mm, ML: ±1.4mm, DV: -4.0mm) injections were performed using a Hamilton microsyringe and a mini-pump (Hamilton, Reno, NV, USA). For induction of an AD model, 20 μl of aggregated Aβ_1-42_ (1 μg/μl Sigma Aldrich, USA, Cat.# SCP0038) at a rate of 60 μl/hr was infused into bilateral LV (10 μl per ventricle). The syringe was removed 5 min after injection. The sham group received the same volume of the sterile vehicle solution. This day was designated as day 0. After surgery, the scalp was sutured, and sulfamethoxazole was sprinkled on the wound to prevent infection. In addition, penicillin (40,000U) was injected intramuscularly into the gluteus once a day for three days ([Kantar Gok et al., 2018](#_ENREF_23); [Wong et al., 2016](#_ENREF_86)). Buprenorphine (0.05mg/kg q12 hours for three days, subcutaneously; Sigma-Aldrich, St. Louis, MO, USA) was used for postoperative analgesia. On day seven after Aβ or vehicle i.c.v. infusion, rats under general anesthesia were subjected to implantation of Alzet osmotic minipumps (#2004, Durect, Cupertino, CA, USA) containing vehicle solution or N-MSC-CM (6 μg/ 6 μl/ day for 28 days of total 168 μg per rat) or H-MSC-CM (6 μg/ 6 μl/ day for 28 days of total 168 μg per rat) ([Wu et al., 2014](#_ENREF_87)) subcutaneously for 28 days. Animals were euthanized with sodium pentobarbital (120 mg/kg, i.p.; Sigma-Aldrich, St. Louis, MO, USA) at 35 d after surgery.

**3. Aβ_1-42_ oligomerization**

Aβ_1-42_ was obtained from Anaspec ( Cat.# AS-20276, Fremont, CA*,* USA), and lyophilized Aβ peptides were prepared by reconstituting the powder in 1% ammonium hydroxide (NH_4_OH) in stocks according to the manufacturer's instructions and previous reports ([Manassero et al., 2016](#_ENREF_40)). The peptide cannot be stored long term in 1% NH_4_OH; therefore, it is important to immediately dilute this solution with 1X PBS to obtain a 30 uM working solution, then subject to oligomerization. For oligomerization, Aβ peptides were incubated at 4℃ for 24 h without shaking and then aliquoted to store at -80 °C until use.

**4. MSC Conditioned medium preparation**

Commercially available human bone marrow mesenchymal stem cells (BMSC, Lonza, Basel, Switzerland) were cultured at an initial density of 5 x 10^5^ in 10 mL Petri dishes and then transferred to T-75 flasks when they had reached confluency. For hypoxic culture, MSCs seeded in a density of 4x10^6^ per 15 cm dish were cultured overnight and then switched to DMEM medium without FBS in 1% O_2_ hypoxia chamber controlled by ProOxC system balanced with 5% CO_2_/95% N_2_ ( Biospherix, Redfield, NY, USA ) for 24h at 37℃. Following incubation, the medium was collected and centrifuged at 1000 xg for 10 min at 4℃. The supernatant was concentrated by Amicon Ultra-15 centrifugal filter units ( 3 kDa, Millipore ) for medium concentration and determined protein concentration. Similarly, normoxic conditioned medium was cultured under normoxic conditions ( 21% O_2_ ). Conditioned media was prepared from 80% to 90% confluent MSC cultures between passages 2 and 4.

**5. Radial arm maze task**

The protocol for this test has been described before ([Baluchnejadmojarad et al., 2019](#_ENREF_2)). All the rats were trained with 1 trial (1) per day for 5 consecutive days before Aβ_1-42_ injection and (2) per week for 5 consecutive weeks after Aβ_1-42_ injection. Finally, the number of long-term memory (reference memory) errors and short-term memory (working memory) errors were counted ([Baluchnejadmojarad et al., 2019](#_ENREF_2)).

**6. Rotarod motor coordination test**

An automated accelerating rotarod apparatus (Rota-rod/RS, PanLab Harvard Apparatus, MA, USA) was used to examine the possible defects in neuromuscular coordination and balance that might occur on the Aβ_1-42_ treated rats ([Huang et al., 2011](#_ENREF_18)).

**7. Passive avoidance test**

Cognitive function was assessed by use of a step-through passive avoidance task on the one day before and every 7 days after surgery as detailed previously ([Abulfadl et al., 2018](#_ENREF_1); [Shahidi et al., 2017](#_ENREF_68)).

**8. Histological analyses**

At day 35 post-surgery, rats were euthanized using an overdose of Zoletil and intracardially perfused with 4% paraformaldehyde in phosphate-buffered saline (Sigma-Aldrich). Following perfusion, the rats' eyes were removed, immersed in 4% paraformaldehyde overnight, and then embedded in paraffin. Five-μm-thick serial sections were taken at 100-μm intervals. Ten sections of each eye were incubated with H & E reagents for cell morphological evaluation. The slides were observed under a light upright microscope (Carl Zeiss, Jena, Germany) by a pathologist blinded to the study design. A digital camera linked to a computer running Axioscope version 4 (Carl Zeiss) was used to capture images. For all eyes, retinal images were obtained at the same distance (1500 μm) from the optic nerve.

**4.9 Immunofluorescence staining (**[**Hsu et al., 2020**](#_ENREF_17)**)**

Retinal sections were mounted onto silane-coated slides (MUTO PURE CHEMICALS Co. Ltd., Tokyo, Japan) and blocked and permeabilized with 0.03 % Triton X-100 (Sigma-Alrich) and 10 % goat serum for 1 h. The sections were then incubated with mouse monoclonal anti-neuronal nuclei (NeuN) antibody (1:200, #MAB377, Merck Millipore, Billerica, MA, USA), β-catenin (1:100, #bs-1165R, Bioss Antibodies, MA, USA), and RPE65 (1:100, #A9615, ABclonal Technology, MA, USA) at 4 ^o^C overnight before being incubated with appropriate secondary antibody (Alexa Fluor 488 or 594-conjugated goat anti-mouse or anti-rabbit IgG secondary antibodies; 1:500, #115-545-003, #115-585-003, 115-545-114, #115-585-114, Jackson ImmunoResearch Laboratories, Inc., PA, USA) at room temperature for 1 h. For quantification of neuron degeneration or apoptosis, sections were then incubated with 0.001% Fluoro-Jade B (#AG310, Merck Millipore) solution or were stained with terminal deoxyribonucleotide transferase-mediated dUTP nick end labeling (TUNEL) assay kit (#TAAP01F-100, BioTnA Biotech, Kaohsiung, Taiwan). The sections were subsequently washed with phosphate buffer, and the nuclei were co-stained with 4,6-diamidino-2-phenylindole (DAPI; 1:5000, #D1306 Invitrogen) using 4',6-diamidino-2-phenylindole (DAPI)-staining mounting medium (Vectashield ®Vector Laboratories, Burlingame, CA, USA). Glass coverslips were mounted on the slides using mounting medium. Digital images were captured with a 40x objective (numerical aperture (N .A.) 0.75) and a 100x oil immersion objective (NA 1.4) by an upright fluorescence microscope system (Carl Zeiss Microscopy GmbH, Jena, Germany) with Zen Software (Carl Zeiss). A pathologist counted the percentage of Fluoro-Jade+NeuN/DAPI and TUNEL+NeuN/DAPI double-labeled cells in 6 fields per section in retina (x 400 magnification).

**9. Cell culture**

The human RPE cell line AREP-19 was purchased from the American Type Culture Collection ( Cat no: CRL-2302, ATCC, Manassas, VA ). The cells were cultured in Dulbecco's modified Eagle medium/F-12 ( DMEM/F-12 ) supplemented with 10% (v/v) fetal bovine serum ( FBS ), 2 mM L-glutamine, 100 μg/ml of streptomycin, and 100 U/ml of penicillin.

Human Mesenchymal stem cell ( MSC ) was obtained from Millipore ( Cat no: SCC034, Billerica, MA, USA ). MSC was cultured in low glucose DMEM with 10 % FBS, 2 mM L-glutamine, and 5 ng/ml human fibroblast growth factor-basic (Millipore).

The cells were maintained at 37 °C in a humidified atmosphere of 95% air and 5% CO_2_ . The medium was changed every 3-4 days. All cell culture reagents were purchased from Gibco ( Thermo Fisher Scientific, Inc., Waltham, MA, USA ).

**10. Measurement of cell viability:**

The 3-(4, 5-dimethylthiazol-3-yl)-2,5-diphenyl tetrazolium bromide ( MTT )( Amresco, Solon, OH, USA ) assay was utilized to determine the cell viability. 2.5x10^5^ /well ARPE-19 cells plated in 6-well plates were allowed to adhere for 24 hr. After treatment, 5 mg ⁄ml MTT in PBS was added to each well to a final concentration of 0.5 mg/ml, and the plates were incubated at 37°C for 2hr. The medium was then removed, and the formazan produced was dissolved in 1ml of DMSO. The optical density was then measured at 540 nm using a MultiSkan GO microplate reader ( Thermo Fisher Scientific, MA, USA ). The values are presented as percentages relative to the untreated controls ( defined as 100% survival ).

**11. Western blot analysis**

After treatment, cells were harvested by trypsinization and centrifugation. Total proteins were extracted by the modified RIPA buffer ( 50mM Tris-HCl, pH 7.4, 1% NP-40, 0.25% Na-deoxycholate, 150 mM NaCl, 1mM EDTA ) containing protease and phosphatase inhibitors ( Sigma, St Louis, MO, USA ) and quantified by Bradford method ( Bio-Rad, Hercules, CA, USA ). For blot analysis, protein extracts were boiled for 5 min in loading buffer and separated on SDS-PAGE and transferred to polyvinylidene difluoride membrane ( Pall Corporation, East Hills, NY, USA ) using wet- transfer system ( Bio-rad ). The membranes were blocked in 5 % non-fat milk in PBS containing 0.05% Tween-20 ( PBS-T ) for 1h at room temperature. The membranes were hybridized with total AKT, pAKT ( Ser473 ), total GSK-3β, pGSK-3β ( Ser9 ), SIRT-1, β-catenin ( all from Cell signaling technology, Beverly, MA, USA), ZO-1 ( Abcam, Cambridge, MA, USA ), Occludin ( Thermo Fisher Scientific, MA, USA ), and β-actin ( Santa Cruz Biotechnology, Santa Cruz, CA, USA ) antibodies for overnight at 4℃. After washing with PBS-T, the membranes were continuously incubated with appropriate secondary antibodies coupled to horseradish peroxidase ( Cell signaling technology ) for 1hr at RT. The blots were developed in the ECL Western detection reagents ( PerkinElmer, Waltham, MA, USA ) and exposed to Hyperfilm ECL (GE Healthcare, Piscataway, NJ, USA). Protein bands were scanned and quantified using ImageMaster TotalLab image analysis software (GE Healthcare). The bands of interest were quantified and normalized against β-actin using Image-Pro Plus v.6.0 software (Media Cybernetics, Inc., Rockville, MD, USA).

**12. Immunofluorescence staining of tight junction proteins**

Cells on slides were fixed with 4% paraformaldehyde for 10 min and permeated with 0.1% Triton X-100 in PBS for 5 min at room temperature. The cells were blocked non-specific binding with 5% non-fat milk in PBS for 1h and then incubated with ZO-1 (Abcam Inc., Boston, MA, USA) and Occludin (Thermo Fisher Scientific) antibodies at 4℃ for overnight. After washing with PBS, slides were incubated with appropriate secondary antibodies (Alexa Fluor 488, Thermo Fisher Scientific), followed by staining rhodamine-phalloidin to label F-actin (Thermo Fisher Scientific) and DAPI. All samples were analyzed with an Axio Imager A2 fluorescence microscope (Zeiss).

**13. Enzyme-linked immunosorbent assay (ELISA)**

The level of IL-6 of cell supernatant was determined using the Human IL-6 ELISA OptEIA kit (BD Biosciences, San Jose, CA, USA) according to the manufacturer's instruction. The absorbance was measured at 450 nm by a microplate reader ( Thermo Fisher Scientific ).

**14. LC-MS/MS analysis**

For identification of secretory proteins in conditioned medium, the concentrated cell-conditioned media was diluted by trypsin digestion buffer (final concentration: 1.0% Sodium deoxycholate/ 25 mM Ammonium bicarbonate). Proteins were reduced with 10 mM Dithiothreitol at 56 oC for 30 min and alkylated with 55 mM Iodoacetamide at room temperature in the dark for 30 min. In-solution digestion of proteins was carried out using MS-grade Trypsin Gold (Promega, Madison, WI) overnight at 37°C. Detergent was acidified by 2% Trifluoroacetic acid and was centrifuged at 16,000 x g to remove. The tryptic digests were extracted and dried in a vacuum concentrator at room temperature and then dissolved in 1 μL of 5% Acetonitrile / 0.5% Trifluoroacetic acid. Peptides were separated using an Ultimate system 3000 nanoLC system (Thermo Fisher Scientific, Bremen, Germany) equipped with a 75 μm ID, 25 cm length C18 Acclaim PepMap NanoLC column (Thermo Fisher Scientific, San Jose, CA, USA) packed with 2 μm particles with a pore of 100 Å. Mobile phase A was 0.1% formic acid in the water, and mobile phase B was composed of 100% acetonitrile with 0.1% formic acid. A segmented gradient in 90 min from 2% to 35% solvent B at a flow rate of 300 nl/min and a column temperature of 35°C were used. Intact peptide mass spectra and fragmentation spectra were acquired on a Thermo Scientific™ Orbitrap Fusion™ Lumos™ Tribrid™ Mass Spectrometer (Thermo Fisher Scientific, UK). Mass spectrometry analysis was performed in a data-dependent mode with Full-MS (externally calibrated to a mass accuracy of <5 ppm, and a resolution of 120,000 at m/z=200) followed by HCD-MS/MS of the most intense ions in 3 s. High-energy collision activated dissociation (HCD)-MS/MS (resolution of 15,000) was used to fragment multiply charged ions (Charge state 2-7) within a 1.4 Da isolation window at a normalized collision energy of 32 eV. AGC target at 5e5 and 5e4 was set for MS and MS/MS analysis, respectively, with previously selected ions dynamically excluded for 180 s. Max injection time 50 ms.

**15. Bioinformatic data analysis and protein network modeling**

The MS/MS signal was analyzed by using the MASCOT searching engine ([www.matrixscience.com](http://www.matrixscience.com)). The functional gene enrichment (gene ontology, GO) analysis was performed in the FunRich software using a built-in FunRich database. GO analysis was carried out to explore the possible cellular component, biological process, and molecular functions related to the proteins. Interaction network analysis was performed using STRING (search tool for the Multiple Proteins, <http://string-db.org/>) version 11.0. The analysis was set for high confidence (score 0.7).

**16. Statistical analysis**

The person charged with functional outcome measurements was the only one that was blinded to treatments among those working on animals (single-blind). She used cage and animal codes to recognize individuals and to report repeated measurements on data collection forms. Statistical analyses were performed using GraphPad Prism 7.01 (GraphPad Software Inc., CA, USA). Parameters such as histological scores and the immunofluorescence staining data with non-normal distribution were analyzed by the Kruskal-Wallis test with Dunn's post-hoc test. One-way analysis of variance (ANOVA) with Tukey's post hoc test was used by an investigator blinded to the treatment groups and the ELISA and Western blotting. We performed two-way ANOVA with Tukey's multiple comparisons tests to analyze behavioral performance data. If there was a significant difference, a Student's t-test was used to compare variables for two groups. All data were expressed as mean ± standard deviation (SD). P-values < 0.05 were considered statistically significant.

**References**

Abulfadl, Y. S., El-Maraghy, N. N., Ahmed, A. E., Nofal, S., Abdel-Mottaleb, Y., & Badary, O. A. (2018). Thymoquinone alleviates the experimentally induced Alzheimer's disease inflammation by modulation of TLRs signaling. *Hum Exp Toxicol, 37*(10), 1092-1104. doi:10.1177/0960327118755256

Baluchnejadmojarad, T., Mohamadi-Zarch, S. M., & Roghani, M. (2019). Safranal, an active ingredient of saffron, attenuates cognitive deficits in amyloid β-induced rat model of Alzheimer's disease: underlying mechanisms. *Metab Brain Dis, 34*(6), 1747-1759. doi:10.1007/s11011-019-00481-6

Ben-Yosef, T., & Francomano, C. A. (1999). Characterization of the human talin (TLN) gene: genomic structure, chromosomal localization, and expression pattern. *Genomics, 62*(2), 316-319. doi:10.1006/geno.1999.6019

Breslow, J. L., Ross, D., McPherson, J., Williams, H., Kurnit, D., Nussbaum, A. L., et al. (1982). Isolation and characterization of cDNA clones for human apolipoprotein A-I. *Proc Natl Acad Sci U S A, 79*(22), 6861-6865. doi:10.1073/pnas.79.22.6861

Broze, G. J., Jr. (1995a). Tissue factor pathway inhibitor. *Thromb Haemost, 74*(1), 90-93.

Broze, G. J., Jr. (1995b). Tissue factor pathway inhibitor and the revised theory of coagulation. *Annu Rev Med, 46*, 103-112. doi:10.1146/annurev.med.46.1.103

Chae, H. Z., Robison, K., Poole, L. B., Church, G., Storz, G., & Rhee, S. G. (1994). Cloning and sequencing of thiol-specific antioxidant from mammalian brain: alkyl hydroperoxide reductase and thiol-specific antioxidant define a large family of antioxidant enzymes. *Proc Natl Acad Sci U S A, 91*(15), 7017-7021. doi:10.1073/pnas.91.15.7017

Chakravarthi, S., Jessop, C. E., Willer, M., Stirling, C. J., & Bulleid, N. J. (2007). Intracellular catalysis of disulfide bond formation by the human sulfhydryl oxidase, QSOX1. *Biochem J, 404*(3), 403-411. doi:10.1042/BJ20061510

Chesik, D., De Keyser, J., & Wilczak, N. (2007). Insulin-like growth factor binding protein-2 as a regulator of IGF actions in CNS: implications in multiple sclerosis. *Cytokine Growth Factor Rev, 18*(3-4), 267-278. doi:10.1016/j.cytogfr.2007.04.001

Cosen-Binker, L. I., & Kapus, A. (2006). Cortactin: the gray eminence of the cytoskeleton. *Physiology (Bethesda), 21*, 352-361. doi:10.1152/physiol.00012.2006

Crabos, M., Yamakado, T., Heizmann, C. W., Cerletti, N., Buhler, F. R., & Erne, P. (1991). The calcium binding protein tropomyosin in human platelets and cardiac tissue: elevation in hypertensive cardiac hypertrophy. *Eur J Clin Invest, 21*(5), 472-478. doi:10.1111/j.1365-2362.1991.tb01397.x

Craparo, A., Freund, R., & Gustafson, T. A. (1997). 14-3-3 (epsilon) interacts with the insulin-like growth factor I receptor and insulin receptor substrate I in a phosphoserine-dependent manner. *J Biol Chem, 272*(17), 11663-11669. doi:10.1074/jbc.272.17.11663

De Cristofaro, R., & De Candia, E. (2003). Thrombin domains: structure, function and interaction with platelet receptors. *J Thromb Thrombolysis, 15*(3), 151-163. doi:10.1023/B:THRO.0000011370.80989.7b

Elshourbagy, N. A., Near, J. C., Kmetz, P. J., Wells, T. N., Groot, P. H., Saxty, B. A., et al. (1992). Cloning and expression of a human ATP-citrate lyase cDNA. *Eur J Biochem, 204*(2), 491-499. doi:10.1111/j.1432-1033.1992.tb16659.x

Faure, J., & Dagher, M. C. (2001). Interactions between Rho GTPases and Rho GDP dissociation inhibitor (Rho-GDI). *Biochimie, 83*(5), 409-414. doi:10.1016/s0300-9084(01)01263-9

Hausmann, E. (1967). Cofactor requirements for the enzymatic hydroxylation of lysine in a polypeptide precursor of collagen. *Biochim Biophys Acta, 133*(3), 591-593. doi:10.1016/0005-2795(67)90566-1

Hsu, C. C., Kuo, T. W., Liu, W. P., Chang, C. P., & Lin, H. J. (2020). Calycosin Preserves BDNF/TrkB Signaling and Reduces Post-Stroke Neurological Injury after Cerebral Ischemia by Reducing Accumulation of Hypertrophic and TNF-α-Containing Microglia in Rats. *J Neuroimmune Pharmacol, 15*(2), 326-339. doi:10.1007/s11481-019-09903-9

Huang, T. C., Lu, K. T., Wo, Y. Y., Wu, Y. J., & Yang, Y. L. (2011). Resveratrol protects rats from Aβ-induced neurotoxicity by the reduction of iNOS expression and lipid peroxidation. *PLoS One, 6*(12), e29102. doi:10.1371/journal.pone.0029102

Janciauskiene, S., & Wright, H. T. (1998). Inflammation, antichymotrypsin, and lipid metabolism: autogenic etiology of Alzheimer's disease. *Bioessays, 20*(12), 1039-1046. doi:10.1002/(SICI)1521-1878(199812)20:12<1039::AID-BIES10>3.0.CO;2-Z

Jiang, J., Yu, L., Huang, X., Chen, X., Li, D., Zhang, Y., et al. (2001). Identification of two novel human dynein light chain genes, DNLC2A and DNLC2B, and their expression changes in hepatocellular carcinoma tissues from 68 Chinese patients. *Gene, 281*(1-2), 103-113. doi:10.1016/s0378-1119(01)00787-9

Jin, D. Y., Lyu, M. S., Kozak, C. A., & Jeang, K. T. (1996). Function of 14-3-3 proteins. *Nature, 382*(6589), 308. doi:10.1038/382308a0

Kalsheker, N. A. (1996). Alpha 1-antichymotrypsin. *Int J Biochem Cell Biol, 28*(9), 961-964. doi:10.1016/1357-2725(96)00032-5

Kantar Gok, D., Hidisoglu, E., Ocak, G. A., Er, H., Acun, A. D., & Yargıcoglu, P. (2018). Protective role of rosmarinic acid on amyloid beta 42-induced echoic memory decline: Implication of oxidative stress and cholinergic impairment. *Neurochem Int, 118*, 1-13. doi:10.1016/j.neuint.2018.04.008

Katahira, T., Nakagiri, S., Terada, K., & Furukawa, T. (2010). Secreted factor FAM3C (ILEI) is involved in retinal laminar formation. *Biochem Biophys Res Commun, 392*(3), 301-306. doi:10.1016/j.bbrc.2009.12.180

Khandelwal, A., Crowley, V. M., & Blagg, B. S. J. (2017). Resorcinol-Based Grp94-Selective Inhibitors. *ACS Med Chem Lett, 8*(10), 1013-1018. doi:10.1021/acsmedchemlett.7b00193

Khetarpal, S. A., Zeng, X., Millar, J. S., Vitali, C., Somasundara, A. V. H., Zanoni, P., et al. (2017). A human APOC3 missense variant and monoclonal antibody accelerate apoC-III clearance and lower triglyceride-rich lipoprotein levels. *Nat Med, 23*(9), 1086-1094. doi:10.1038/nm.4390

Kishimoto, Y., Hiraiwa, M., & O'Brien, J. S. (1992). Saposins: structure, function, distribution, and molecular genetics. *J Lipid Res, 33*(9), 1255-1267.

Lamande, S. R., Morgelin, M., Adams, N. E., Selan, C., & Allen, J. M. (2006). The C5 domain of the collagen VI alpha3(VI) chain is critical for extracellular microfibril formation and is present in the extracellular matrix of cultured cells. *J Biol Chem, 281*(24), 16607-16614. doi:10.1074/jbc.M510192200

Lamartine, J., Seri, M., Cinti, R., Heitzmann, F., Creaven, M., Radomski, N., et al. (1997). Molecular cloning and mapping of a human cDNA (PA2G4) that encodes a protein highly homologous to the mouse cell cycle protein p38-2G4. *Cytogenet Cell Genet, 78*(1), 31-35. doi:10.1159/000134621

Lancaster, C. A., Taylor-Harris, P. M., Self, A. J., Brill, S., van Erp, H. E., & Hall, A. (1994). Characterization of rhoGAP. A GTPase-activating protein for rho-related small GTPases. *J Biol Chem, 269*(2), 1137-1142.

Langbein, L., Heid, H. W., Moll, I., & Franke, W. W. (1993). Molecular characterization of the body site-specific human epidermal cytokeratin 9: cDNA cloning, amino acid sequence, and tissue specificity of gene expression. *Differentiation, 55*(1), 57-71. doi:10.1111/j.1432-0436.1993.tb00033.x

Larocque, G., La-Borde, P. J., Clarke, N. I., Carter, N. J., & Royle, S. J. (2020). Tumor protein D54 defines a new class of intracellular transport vesicles. *J Cell Biol, 219*(1). doi:10.1083/jcb.201812044

Lessor, T. J., & Hamburger, A. W. (2001). Regulation of the ErbB3 binding protein Ebp1 by protein kinase C. *Mol Cell Endocrinol, 175*(1-2), 185-191. doi:10.1016/s0303-7207(01)00387-2

Li, G., Yin, Y., Chen, J., Fan, Y., Ma, J., Huang, Y., et al. (2018). Coactosin-like protein 1 inhibits neuronal migration during mouse corticogenesis. *J Vet Sci, 19*(1), 21-26. doi:10.4142/jvs.2018.19.1.21

Liu, L., Watanabe, N., Akatsu, H., & Nishimura, M. (2016). Neuronal expression of ILEI/FAM3C and its reduction in Alzheimer's disease. *Neuroscience, 330*, 236-246. doi:10.1016/j.neuroscience.2016.05.050

Liu, R., & Jin, J. P. (2016). Calponin isoforms CNN1, CNN2 and CNN3: Regulators for actin cytoskeleton functions in smooth muscle and non-muscle cells. *Gene, 585*(1), 143-153. doi:10.1016/j.gene.2016.02.040

Luo, G., Ducy, P., McKee, M. D., Pinero, G. J., Loyer, E., Behringer, R. R., et al. (1997). Spontaneous calcification of arteries and cartilage in mice lacking matrix GLA protein. *Nature, 386*(6620), 78-81. doi:10.1038/386078a0

Lusic, M., Marcello, A., Cereseto, A., & Giacca, M. (2003). Regulation of HIV-1 gene expression by histone acetylation and factor recruitment at the LTR promoter. *Embo j, 22*(24), 6550-6561. doi:10.1093/emboj/cdg631

Maguchi, M., Nishida, W., Kohara, K., Kuwano, A., Kondo, I., & Hiwada, K. (1995). Molecular cloning and gene mapping of human basic and acidic calponins. *Biochem Biophys Res Commun, 217*(1), 238-244. doi:10.1006/bbrc.1995.2769

Manassero, G., Guglielmotto, M., Zamfir, R., Borghi, R., Colombo, L., Salmona, M., et al. (2016). Beta-amyloid 1-42 monomers, but not oligomers, produce PHF-like conformation of Tau protein. *Aging Cell, 15*(5), 914-923. doi:<https://doi.org/10.1111/acel.12500>

Mandle, R. J., Colman, R. W., & Kaplan, A. P. (1976). Identification of prekallikrein and high-molecular-weight kininogen as a complex in human plasma. *Proc Natl Acad Sci U S A, 73*(11), 4179-4183. doi:10.1073/pnas.73.11.4179

Manso, A. M., Li, R., Monkley, S. J., Cruz, N. M., Ong, S., Lao, D. H., et al. (2013). Talin1 has unique expression versus talin 2 in the heart and modifies the hypertrophic response to pressure overload. *J Biol Chem, 288*(6), 4252-4264. doi:10.1074/jbc.M112.427484

Mathieu, C., Li de la Sierra-Gallay, I., Duval, R., Xu, X., Cocaign, A., Leger, T., et al. (2016). Insights into Brain Glycogen Metabolism: THE STRUCTURE OF HUMAN BRAIN GLYCOGEN PHOSPHORYLASE. *J Biol Chem, 291*(35), 18072-18083. doi:10.1074/jbc.M116.738898

Meindl, A., Carvalho, M. R., Herrmann, K., Lorenz, B., Achatz, H., Lorenz, B., et al. (1995). A gene (SRPX) encoding a sushi-repeat-containing protein is deleted in patients with X-linked retinitis pigmentosa. *Hum Mol Genet, 4*(12), 2339-2346. doi:10.1093/hmg/4.12.2339

Mendivil, C. O., Zheng, C., Furtado, J., Lel, J., & Sacks, F. M. (2010). Metabolism of very-low-density lipoprotein and low-density lipoprotein containing apolipoprotein C-III and not other small apolipoproteins. *Arterioscler Thromb Vasc Biol, 30*(2), 239-245. doi:10.1161/ATVBAHA.109.197830

Metral, S., Machnicka, B., Bigot, S., Colin, Y., Dhermy, D., & Lecomte, M. C. (2009). AlphaII-spectrin is critical for cell adhesion and cell cycle. *J Biol Chem, 284*(4), 2409-2418. doi:10.1074/jbc.M801324200

Moren, A., Olofsson, A., Stenman, G., Sahlin, P., Kanzaki, T., Claesson-Welsh, L., et al. (1994). Identification and characterization of LTBP-2, a novel latent transforming growth factor-beta-binding protein. *J Biol Chem, 269*(51), 32469-32478.

Morimoto, S., Yamamoto, Y., O'Brien, J. S., & Kishimoto, Y. (1990). Distribution of saposin proteins (sphingolipid activator proteins) in lysosomal storage and other diseases. *Proc Natl Acad Sci U S A, 87*(9), 3493-3497. doi:10.1073/pnas.87.9.3493

Munger, J. S., Harpel, J. G., Gleizes, P. E., Mazzieri, R., Nunes, I., & Rifkin, D. B. (1997). Latent transforming growth factor-beta: structural features and mechanisms of activation. *Kidney Int, 51*(5), 1376-1382. doi:10.1038/ki.1997.188

Munroe, P. B., Olgunturk, R. O., Fryns, J. P., Van Maldergem, L., Ziereisen, F., Yuksel, B., et al. (1999). Mutations in the gene encoding the human matrix Gla protein cause Keutel syndrome. *Nat Genet, 21*(1), 142-144. doi:10.1038/5102

Nelson, J., McFerran, N. V., Pivato, G., Chambers, E., Doherty, C., Steele, D., et al. (2008). The 67 kDa laminin receptor: structure, function and role in disease. *Biosci Rep, 28*(1), 33-48. doi:10.1042/BSR20070004

Newgard, C. B., Littman, D. R., van Genderen, C., Smith, M., & Fletterick, R. J. (1988). Human brain glycogen phosphorylase. Cloning, sequence analysis, chromosomal mapping, tissue expression, and comparison with the human liver and muscle isozymes. *J Biol Chem, 263*(8), 3850-3857.

Nimpf, J., Wurm, H., & Kostner, G. M. (1985). Interaction of beta 2-glycoprotein-I with human blood platelets: influence upon the ADP-induced aggregation. *Thromb Haemost, 54*(2), 397-401.

Nissen, S. E., Tsunoda, T., Tuzcu, E. M., Schoenhagen, P., Cooper, C. J., Yasin, M., et al. (2003). Effect of recombinant ApoA-I Milano on coronary atherosclerosis in patients with acute coronary syndromes: a randomized controlled trial. *JAMA, 290*(17), 2292-2300. doi:10.1001/jama.290.17.2292

Niu, J. X., Zhang, W. J., Ye, L. Y., Wu, L. Q., Zhu, G. J., Yang, Z. H., et al. (2007). The role of adhesion molecules, alpha v beta 3, alpha v beta 5 and their ligands in the tumor cell and endothelial cell adhesion. *Eur J Cancer Prev, 16*(6), 517-527. doi:10.1097/CEJ.0b013e3280145c00

Odh, G., Hindemith, A., Rosengren, A. M., Rosengren, E., & Rorsman, H. (1993). Isolation of a new tautomerase monitored by the conversion of D-dopachrome to 5,6-dihydroxyindole. *Biochem Biophys Res Commun, 197*(2), 619-624. doi:10.1006/bbrc.1993.2524

Ould-Abeih, M. B., Petit-Topin, I., Zidane, N., Baron, B., & Bedouelle, H. (2012). Multiple folding states and disorder of ribosomal protein SA, a membrane receptor for laminin, anticarcinogens, and pathogens. *Biochemistry, 51*(24), 4807-4821. doi:10.1021/bi300335r

Palmgren, S., Vartiainen, M., & Lappalainen, P. (2002). Twinfilin, a molecular mailman for actin monomers. *J Cell Sci, 115*(Pt 5), 881-886.

Peter, B., Polyansky, A. A., Fanucchi, S., & Dirr, H. W. (2014). A Lys-Trp cation-pi interaction mediates the dimerization and function of the chloride intracellular channel protein 1 transmembrane domain. *Biochemistry, 53*(1), 57-67. doi:10.1021/bi401433f

Provost, P., Doucet, J., Stock, A., Gerisch, G., Samuelsson, B., & Radmark, O. (2001). Coactosin-like protein, a human F-actin-binding protein: critical role of lysine-75. *Biochem J, 359*(Pt 2), 255-263. doi:10.1042/0264-6021:3590255

Reis, A., Hennies, H. C., Langbein, L., Digweed, M., Mischke, D., Drechsler, M., et al. (1994). Keratin 9 gene mutations in epidermolytic palmoplantar keratoderma (EPPK). *Nat Genet, 6*(2), 174-179. doi:10.1038/ng0294-174

Rothnagel, J. A., Dominey, A. M., Dempsey, L. D., Longley, M. A., Greenhalgh, D. A., Gagne, T. A., et al. (1992). Mutations in the rod domains of keratins 1 and 10 in epidermolytic hyperkeratosis. *Science, 257*(5073), 1128-1130. doi:10.1126/science.257.5073.1128

Sahab, Z. J., Hall, M. D., Me Sung, Y., Dakshanamurthy, S., Ji, Y., Kumar, D., et al. (2011). Tumor suppressor RARRES1 interacts with cytoplasmic carboxypeptidase AGBL2 to regulate the alpha-tubulin tyrosination cycle. *Cancer Res, 71*(4), 1219-1228. doi:10.1158/0008-5472.CAN-10-2294

Schild, H., & Rammensee, H. G. (2000). gp96--the immune system's Swiss army knife. *Nat Immunol, 1*(2), 100-101. doi:10.1038/77770

Schroder, W. A., Major, L., & Suhrbier, A. (2011). The role of SerpinB2 in immunity. *Crit Rev Immunol, 31*(1), 15-30. doi:10.1615/critrevimmunol.v31.i1.20

Scully, R., & Xie, A. (2013). Double strand break repair functions of histone H2AX. *Mutat Res, 750*(1-2), 5-14. doi:10.1016/j.mrfmmm.2013.07.007

Segers, K., Dahlback, B., & Nicolaes, G. A. (2007). Coagulation factor V and thrombophilia: background and mechanisms. *Thromb Haemost, 98*(3), 530-542.

Shahidi, S., Zargooshnia, S., Asl, S. S., Komaki, A., & Sarihi, A. (2017). Influence of N-acetyl cysteine on beta-amyloid-induced Alzheimer's disease in a rat model: A behavioral and electrophysiological study. *Brain Res Bull, 131*, 142-149. doi:10.1016/j.brainresbull.2017.04.001

Stormorken, H. (2003). The discovery of factor V: a tricky clotting factor. *J Thromb Haemost, 1*(2), 206-213. doi:10.1046/j.1538-7836.2003.00043.x

Strik, M. C., Wolbink, A., Wouters, D., Bladergroen, B. A., Verlaan, A. R., van Houdt, I. S., et al. (2004). Intracellular serpin SERPINB6 (PI6) is abundantly expressed by human mast cells and forms complexes with beta-tryptase monomers. *Blood, 103*(7), 2710-2717. doi:10.1182/blood-2003-08-2981

Sun, J., Coughlin, P., Salem, H. H., & Bird, P. (1995). Production and characterization of recombinant human proteinase inhibitor 6 expressed in Pichia pastoris. *Biochim Biophys Acta, 1252*(1), 28-34. doi:10.1016/0167-4838(95)00108-7

Susalka, S. J., Nikulina, K., Salata, M. W., Vaughan, P. S., King, S. M., Vaughan, K. T., et al. (2002). The roadblock light chain binds a novel region of the cytoplasmic Dynein intermediate chain. *J Biol Chem, 277*(36), 32939-32946. doi:10.1074/jbc.M205510200

Svitkina, T. M., Verkhovsky, A. B., & Borisy, G. G. (1996). Plectin sidearms mediate interaction of intermediate filaments with microtubules and other components of the cytoskeleton. *J Cell Biol, 135*(4), 991-1007. doi:10.1083/jcb.135.4.991

Takaluoma, K., Hyry, M., Lantto, J., Sormunen, R., Bank, R. A., Kivirikko, K. I., et al. (2007). Tissue-specific changes in the hydroxylysine content and cross-links of collagens and alterations in fibril morphology in lysyl hydroxylase 1 knock-out mice. *J Biol Chem, 282*(9), 6588-6596. doi:10.1074/jbc.M608830200

Tamura, T., Lee, D. H., Osaka, F., Fujiwara, T., Shin, S., Chung, C. H., et al. (1991). Molecular cloning and sequence analysis of cDNAs for five major subunits of human proteasomes (multi-catalytic proteinase complexes). *Biochim Biophys Acta, 1089*(1), 95-102. doi:10.1016/0167-4781(91)90090-9

Thorpe, C., Hoober, K. L., Raje, S., Glynn, N. M., Burnside, J., Turi, G. K., et al. (2002). Sulfhydryl oxidases: emerging catalysts of protein disulfide bond formation in eukaryotes. *Arch Biochem Biophys, 405*(1), 1-12. doi:10.1016/s0003-9861(02)00337-5

Tkachenko, A. V., Buchman, V. L., Bliskovsky, V. V., Shvets Yu, P., & Kisselev, L. L. (1992). Exons I and VII of the gene (Ker10) encoding human keratin 10 undergo structural rearrangements within repeats. *Gene, 116*(2), 245-251. doi:10.1016/0378-1119(92)90521-p

Ursitti, J. A., Petrich, B. G., Lee, P. C., Resneck, W. G., Ye, X., Yang, J., et al. (2007). Role of an alternatively spliced form of alphaII-spectrin in localization of connexin 43 in cardiomyocytes and regulation by stress-activated protein kinase. *J Mol Cell Cardiol, 42*(3), 572-581. doi:10.1016/j.yjmcc.2006.11.018

Valiente, M., Obenauf, A. C., Jin, X., Chen, Q., Zhang, X. H., Lee, D. J., et al. (2014). Serpins promote cancer cell survival and vascular co-option in brain metastasis. *Cell, 156*(5), 1002-1016. doi:10.1016/j.cell.2014.01.040

Vartiainen, M., Ojala, P. J., Auvinen, P., Peranen, J., & Lappalainen, P. (2000). Mouse A6/twinfilin is an actin monomer-binding protein that localizes to the regions of rapid actin dynamics. *Mol Cell Biol, 20*(5), 1772-1783. doi:10.1128/mcb.20.5.1772-1783.2000

Vikesaa, J., Hansen, T. V., Jonson, L., Borup, R., Wewer, U. M., Christiansen, J., et al. (2006). RNA-binding IMPs promote cell adhesion and invadopodia formation. *Embo j, 25*(7), 1456-1468. doi:10.1038/sj.emboj.7601039

von Bulow, M., Rackwitz, H. R., Zimbelmann, R., & Franke, W. W. (1997). CP beta3, a novel isoform of an actin-binding protein, is a component of the cytoskeletal calyx of the mammalian sperm head. *Exp Cell Res, 233*(1), 216-224. doi:10.1006/excr.1997.3564

Wang, C. L. (2001). Caldesmon and smooth-muscle regulation. *Cell Biochem Biophys, 35*(3), 275-288. doi:10.1385/cbb:35:3:275

Wilton, S. D., Lim, L., Dorosz, S. D., Gunn, H. C., Eyre, H. J., Callen, D. F., et al. (1996). Assignment of the human alpha-tropomyosin gene TPM4 to band 19p13.1 by fluorescence in situ hybridization. *Cytogenet Cell Genet, 72*(4), 294-296. doi:10.1159/000134206

Wolf, E., Lahm, H., Wu, M., Wanke, R., & Hoeflich, A. (2000). Effects of IGFBP-2 overexpression in vitro and in vivo. *Pediatr Nephrol, 14*(7), 572-578. doi:10.1007/s004670000362

Wong, R. S., Cechetto, D. F., & Whitehead, S. N. (2016). Assessing the Effects of Acute Amyloid β Oligomer Exposure in the Rat. *Int J Mol Sci, 17*(9). doi:10.3390/ijms17091390

Wu, C. R., Lin, H. C., & Su, M. H. (2014). Reversal by aqueous extracts of Cistanche tubulosa from behavioral deficits in Alzheimer's disease-like rat model: relevance for amyloid deposition and central neurotransmitter function. *BMC Complement Altern Med, 14*, 202. doi:10.1186/1472-6882-14-202

Xu, X., Kedlaya, R., Higuchi, H., Ikeda, S., Justice, M. J., Setaluri, V., et al. (2010). Mutation in archain 1, a subunit of COPI coatomer complex, causes diluted coat color and Purkinje cell degeneration. *PLoS Genet, 6*(5), e1000956. doi:10.1371/journal.pgen.1000956

**Supplemental Table 1: Normoxic mesenchymal stem cell-conditioned medium (N-CM) protein identifications by using MS/MS**

| **No.** | **Gene Symbol** | **Protein name** | **Accession number** | **Protein functions** |
| --- | --- | --- | --- | --- |
| 1 | COL6A3 | collagen, type VI, alpha 3 | NP_004360.2 | An extracellular matrix binding protein , which is an intricate lattice that forms in the space between cells and provides structural support.([Lamande et al., 2006](#_ENREF_28)) |
| 2 | IGFBP2 | insulin-like growth factor binding protein 2, 36kDa | NP_000588.3 | Regulation the growth promoting effects of the IGFs in CNS. Implications in multiple sclerosis.([Chesik et al., 2007](#_ENREF_9); [Wolf et al., 2000](#_ENREF_85)) |
| 3 | YWHAE | tyrosine 3-monooxygenase/ tryptophan 5-monooxygenase activation protein, epsilon | NP_006752.1 | Regulate a variety of processes including cell division and sensitivity to insulin, a hormone that helps control blood sugar levels.([Craparo et al., 1997](#_ENREF_12); [Jin et al., 1996](#_ENREF_21)) |
| 4 | APOC3 | apolipoprotein C-III | NP_000031.1 | Promote the secretion of VLDL1 and inhibit lipoprotein lipase enzyme activity. Association with low plasma triglyceride levels , ischemic cardiovascular disease and hyperalphalipoproteinemia.([Khetarpal et al., 2017](#_ENREF_26); [Mendivil et al., 2010](#_ENREF_45)) |
| 5 | SERPINB2 | serpin peptidase inhibitor, clade B (ovalbumin), member 2 | NP_002566.1 | A coagulation factor that inactivates tPA and urokinase. Implications in cancer metastasis depend on cancer type and location.([Schroder et al., 2011](#_ENREF_65); [Valiente et al., 2014](#_ENREF_79)) |
| 6 | ACLY | ATP citrate lyase | NP_001087.2 | Catalyzes the cleavage of citrate into oxaloacetate and acetyl-CoA, the latter serving as common substrate for de novo cholesterol and fatty acid synthesis.([Elshourbagy et al., 1992](#_ENREF_14)) |
| 7 | ARHGDIA | Rho GDP dissociation inhibitor (GDI) alpha | NP_004300.1 | Controls Rho proteins homeostasis and regulates the GDP/GTP exchange reaction of the Rho proteins.([Faure et al., 2001](#_ENREF_15)) |
| 8 | FAM3C | family with sequence similarity 3, member C | NP_055703.1 | Regulates in retinal laminar formationand promotes epithelial to mesenchymal transition. A reduced FAM3C level associated to the onset of sporadic Alzheimer's disease (AD).([Katahira et al., 2010](#_ENREF_24); [L. Liu et al., 2016](#_ENREF_35)) |
| 9 | RARRES1 | retinoic acid receptor responder (tazarotene induced) 1 | NP_002879.2 | Inhibits the cytoplasmic carboxypeptidase AGBL2 and regulates the alpha-tubulin tyrosination cycle.([Sahab et al., 2011](#_ENREF_63)) |
| 10 | MGP | matrix Gla protein | NP_000891.2 | A physiological inhibitor of ectopic tissue calcification.Mutations in this gene cause Keutel syndrome in human patients.([Luo et al., 1997](#_ENREF_37); [Munroe et al., 1999](#_ENREF_50)) |
| 11 | PLOD1 | Procollagen-lysine, 2-oxoglutarate 5-dioxygenase 1 | NP_000293.2 | Catalyzes hydroxylation of lysine residues in collagen alpha chains and is required for normal assembly and cross-linkling of collagen fibrils.([Hausmann, 1967](#_ENREF_16); [Takaluoma et al., 2007](#_ENREF_74)) |
| 12 | CALD1 | Caldesmon 1 | NP_004333.1 | Regulation of actomyosin interactions in smooth muscle and nonmuscle cells. A bridge between myosin and actin filaments.([Wang, 2001](#_ENREF_83)) |
| 13 | CAPZB | Capping protein (actin filament) muscle Z-line, beta | NP_004921.1 | Regulates growth of the actin filament by capping the barbed end of growing actin filaments.([von Bulow et al., 1997](#_ENREF_82)) |
| 14 | PSAP | Prosaposin | NP_002769.1 | Involved in the development of the nervous system and the reproductive system. Associated with Gaucher disease, Tay–Sachs disease, and metachromatic leukodystrophy. ([Kishimoto et al., 1992](#_ENREF_27); [Morimoto et al., 1990](#_ENREF_48)) |
| 15 | KNG1 | Kininogen 1 | NP_000884.1 | A circulating plasma protein which participates in the initiation of blood coagulation and generation of the vasodilator bradykinin via the kallikrein-kinin system.([Mandle et al., 1976](#_ENREF_41)) |
| 16 | SRPX | Sushi-repeat containing protein, X-linked | NP_006298.1 | Deleted in patients with X-linked retinitis pigmentosa.([Meindl et al., 1995](#_ENREF_44)) |
| 17 | RPSA | Ribosomal protein SA | NP_002286.2 | Instructions for making ribosomal protein SA to form ribosomes. Cell surface receptor for laminin. Plays a role in cell adhesion to the basement membrane.([Nelson et al., 2008](#_ENREF_51); [Ould-Abeih et al., 2012](#_ENREF_57)) |
| 18 | HSP90B1 | Heat shock protein 90kDa beta (Grp94), member 1 | NP_003290.1 | Molecular chaperone that functions in the processing and transport of secreted proteins. A target for treatment of a plethora of diseases.([Khandelwal et al., 2017](#_ENREF_25); [Schild et al., 2000](#_ENREF_64)) |
| 19 | H2AFX | H2A histone family, member X | NP_002096.1 | A type of histone protein. Responsible for the nucleosome structure of the chromosomal fiber in eukaryotes.([Scully et al., 2013](#_ENREF_66)) |
| 20 | APOA1 | Apolipoprotein A-I | NP_000030.1 | Participates in the reverse transport of cholesterol from tissues to the liver. A cofactor for the lecithin cholesterol acyltransferase (LCAT). Activity associated with high HDL-C and protection from heart disease.([Breslow et al., 1982](#_ENREF_4); [Nissen et al., 2003](#_ENREF_54)) |
| 21 | EDIL3 | EGF-like repeats and discoidin I-like domains 3 | NP_005702.3 | An integrin ligand that plays an important role in mediating angiogenesis and vessel wall remodeling.([Niu et al., 2007](#_ENREF_55)) |
| 22 | ARCN1 | Archain 1 | NP_001646.2 | An intracellular protein. Similarities to heat shock proteins and clathrin-associated proteins and involved in vesicle structure or trafficking.([Xu et al., 2010](#_ENREF_88)) |

**Supplemental Table 2: Hypoxic mesenchymal stem cell-conditioned medium (H-CM) protein identifications by using MS/MS**

| **No.** | **Gene Symbol** | **Protein name** | **Accession number** | **Protein functions** |
| --- | --- | --- | --- | --- |
| 1 | QSOX1 | quiescin Q6 sulfhydryl oxidase 1 | NP_002817.2 | Catalyzes the oxidation of sulfhydryl groups in peptide and protein thiols to disulfides with the reduction of oxygen to hydrogen peroxide. ([Chakravarthi et al., 2007](#_ENREF_8); [Thorpe et al., 2002](#_ENREF_76)) |
| 2 | TPM4 | tropomyosin 4 | NP_003281.1 | Binds to actin filaments in muscle and non-muscle cells and regulates vertebrate striated muscle contraction.([Crabos et al., 1991](#_ENREF_11); [Wilton et al., 1996](#_ENREF_84)) |
| 3 | TLN1 | talin 1 | NP_006280.3 | Mediates cell-cell adhesion via the linkage of integrins to the actin cytoskeleton. Altered expression observed in patients with heart failure.([Ben-Yosef et al., 1999](#_ENREF_3); [Manso et al., 2013](#_ENREF_42)) |
| 4 | KRT10 | keratin 10, type I | NP_000412.3 | Plays a role in the establishment of the epidermal barrier on plantar skin. Mutations in this gene are associated with epidermolytic hyperkeratosis.([Rothnagel et al., 1992](#_ENREF_62); [Tkachenko et al., 1992](#_ENREF_77)) |
| 5 | COTL1 | coactosin-like F-actin binding protein 1 | NP_066972.1 | Regulates the actin cytoskeleton and involves in the regulation of neuronal migration and morphogenesis.([Li et al., 2018](#_ENREF_34); [Provost et al., 2001](#_ENREF_60)) |
| 6 | HIST1H2AI | histone cluster 1, H2ai | NP_003500.1 | Basic nuclear proteins that are responsible for the nucleosome structure in eukaryotes.([Lusic et al., 2003](#_ENREF_38)) |
| 7 | KRT1 | keratin 1, type II | NP_006112.3 | Regulate the activity of kinases via binding to integrin beta-1 and the receptor of activated protein C kinase 1.([Rothnagel et al., 1992](#_ENREF_62)) |
| 8 | KRT9 | keratin 9, type I | NP_000217.2 | An intermediate filament chain expressed only in the terminally differentiated epidermis of palms and soles. Mutations in this gene cause epidermolytic palmoplantar keratoderma.([Langbein et al., 1993](#_ENREF_31); [Reis et al., 1994](#_ENREF_61)) |
| 9 | F2 | coagulation factor II (thrombin) | NP_000497.1 | A serine protease that converts soluble fibrinogen into insoluble strands of fibrin. Functions in blood homeostasis, inflammation and wound healing.([De Cristofaro et al., 2003](#_ENREF_13)) |
| 10 | SERPINB6 | serpin peptidase inhibitor, clade B (ovalbumin), member 6 | NP_004559.4 | Regulation of serine proteinases present in the brain or extravasated from the blood. Inhibitor of cathepsin G, kallikrein-8 and thrombin.([Strik et al., 2004](#_ENREF_70); [Sun et al., 1995](#_ENREF_71)) |
| 11 | F5 | coagulation factor V (proaccelerin, labile factor) | NP_000121.2 | Central regulator of hemostasis. a critical cofactor for the prothrombinase activity of factor Xa.([Segers et al., 2007](#_ENREF_67); [Stormorken, 2003](#_ENREF_69)) |
| 12 | CD44 | CD44 molecule ( Indian blood group) | NP_000601.3 | Cell-surface receptor that plays a role in cell-cell interactions, cell adhesion and migration.([Vikesaa et al., 2006](#_ENREF_81)) |
| 13 | SERPINA3 | serpin peptidase inhibitor, clade A (alpha-1 antiproteinase, antitrypsin), member 3 | NP_001076.2 | Inhibit neutrophil cathepsin G and mast cell chymase. Associated with Parkinson disease and chronic obstructive pulmonary disease.([Janciauskiene et al., 1998](#_ENREF_19); [Kalsheker, 1996](#_ENREF_22)) |
| 14 | APOH | apolipoprotein H (beta-2-glycoprotein I) | NP_000033.2 | Alters adenosine diphosphate (ADP)-mediated agglutination of platelets as an anticoagulation in serum.([Nimpf et al., 1985](#_ENREF_53)) |
| 15 | TPD52L2 | tumor protein D52-like 2 | NP_003279.2 | Transfer proteins between different cellular compartments as a membrane traffic.([Larocque et al., 2020](#_ENREF_32)) |
| 16 | PA2G4 | proliferation-associated 2G4, 38kDa | NP_006182.2 | An ErbB3-binding protein 1 that is involved in ribosome assembly and the regulation of intermediate and late steps of rRNA processing.([Lamartine et al., 1997](#_ENREF_29); [Lessor et al., 2001](#_ENREF_33)) |
| 17 | LTBP2 | latent transforming growth factor beta binding protein 2 | NP_000419.1 | Plays an integral structural role in elastic-fiber architectural organization and/or assembly.([Moren et al., 1994](#_ENREF_47); [Munger et al., 1997](#_ENREF_49)) |
| 18 | SPTAN1 | spectrin, alpha, non-erythrocytic 1 | NP_003118.2 | A cytoskeletal proteins which are involved in actin crosslinking, cell adhesion, intercellular communication and cell cycle regulation.([Metral et al., 2009](#_ENREF_46); [Ursitti et al., 2007](#_ENREF_78)) |
| 19 | PSMA1 | proteasome (prosome, macropain) subunit, alpha type, 1 | NP_002777.1 | Component of the 20S core proteasome complex involved in the proteolytic degradation of most intracellular proteins.([Tamura et al., 1991](#_ENREF_75)) |
| 20 | PRDX2 | peroxiredoxin 2 | NP_005800.3 | Plays a role in cell protection against oxidative stress by detoxifying peroxides and as sensor of hydrogen peroxide-mediated signaling.([Chae et al., 1994](#_ENREF_7)) |
| 21 | PYGB | phosphorylase, glycogen; brain | NP_002853.2 | A glycogen phosphorylase that regulates glycogen mobilization and contributes to the regulation of carbohydrate metabolism.([Mathieu et al., 2016](#_ENREF_43); [Newgard et al., 1988](#_ENREF_52)) |
| 22 | PLEC | plectin | NP_000436.2 | Acts as a link between the three main components of the cytoskeleton: actin microfilaments, microtubules and intermediate filaments.([Svitkina et al., 1996](#_ENREF_73)) |
| 23 | TWF1 | twinfilin actin binding protein 1 | NP_002813.3 | An actin-binding protein involved in motile and morphological processes.([Palmgren et al., 2002](#_ENREF_58); [Vartiainen et al., 2000](#_ENREF_80)) |
| 24 | DDT | D-dopachrome tautomerase | NP_001346.1 | Tautomerization of D-dopachrome with decarboxylation to give 5,6-dihydroxyindole (DHI).([Odh et al., 1993](#_ENREF_56)) |
| 25 | ARHGAP1 | Rho GTPase activating protein 1 | NP_004299.1 | GTPase activator for the Rho, Rac and Cdc42 proteins, converting them to the putatively inactive GDP-bound state.([Lancaster et al., 1994](#_ENREF_30)) |
| 26 | CNN3 | calponin 3, acidic | NP_001830.1 | Thin filament-associated protein is implicated in modulation of smooth muscle contraction.([R. Liu et al., 2016](#_ENREF_36); [Maguchi et al., 1995](#_ENREF_39)) |
| 27 | CLIC1 | chloride intracellular channel 1 | NP_001279.2 | Stabilization of cell membrane potential, transepithelial transport, maintenance of intracellular pH, and regulation of cell volume.([Peter et al., 2014](#_ENREF_59)) |
| 28 | DYNLRB1 | dynein, light chain, roadblock-type 1 | NP_054902.1 | A cytoplasmic protein that is capable of binding intermediate chain proteins.([Jiang et al., 2001](#_ENREF_20); [Susalka et al., 2002](#_ENREF_72)) |
| 29 | TFPI | tissue factor pathway inhibitor (lipoprotein-associated coagulation inhibitor) | NP_006278.1 | Possesses an antithrombotic action and associates with lipoproteins in plasma.([Broze, 1995a](#_ENREF_5), [1995b](#_ENREF_6)) |
| 30 | CTTN | cortactin | NP_005222.2 | Promotes polymerization and rearrangement of the actin cytoskeleton.([Cosen-Binker et al., 2006](#_ENREF_10)) |

**Figure S1: Experimental design.** In the *in vivo* study, SD rats were allowed to rest and acclimate to the environment for at least seven days. Rats were subjected to four days of spatial learning, passive avoidance learning, and rotarod running and pretest. On day 0, the rat was anesthetized and placed onto a stereotaxic apparatus. Bilateral intracerebroventricular (i.c.v.) injections were performed using a Hamilton syringe.

Rats were received bilateral i.c.v. injection of Aβ_1-42_ 10 μl/ventricle under anesthetized. After seven days of post-Aβ injection, rats have implanted Alzet minipump into the dorsal subcutaneous under anesthesia. After surgery, rats were allowed to recover for one week before behavioral assessments began. The radial maze, passive avoidance, and rotarod tests were performed at days 14, 21, 28, and 35 post-Aβ injection or sham operation. The eyes were removed for the H & E staining and immunofluorescence staining. In the *in vitro* study, the ARPE19 cell line was added Aβ_1-42_ (30 μM) or vehicle for 24 hours and then removed the medium and added N-CM (250 μg/ml) or H-CM (250 μg/ml). After 24 hours of incubation with N-CM or H-CM or vehicle, cells and supernatant were collected for cell viability (by MTT assay), apoptosis (by flow cytometry), tight junction protein integrity (IF stain and Western blot), SIRT1/pAKT/pGSK3β/β-catenin pathway (by Western blot), and protein identification (by MS/MS, gene ontology, and String analysis).

**Figure S2.** **FluoroJade B (FJB) staining of the rat retina (left and right side) from different groups of rats.** (A) Representative images (400 X magnification) of NeuN (neuron marker, red) expression coexisted with signals from FJB (green) in the GCL (arrows) from a Sham+Veh, an Aβ+Veh, an Aβ+N-CM, and an Aβ+H-CM rat. Blue signals from DAPI showed nuclear staining. Superimposition of red and green signals produced a yellow signal indicating the presence of neuron degeneration (arrowheads). The scale bar represents 20 μm and 50 μm. (B) Histograms showing the average numbers of FJB-positive neurons in the GCL of the retina. Values are shown as means±SD (n=6 of each group). *p<0.05, compared with Sham+Veh group; +p<0.05, compared with Aβ+Veh group.

**Figure S3: Analysis of Retinal Pigment Epithelium (RPE) apoptosis after Aβ_1-42_ injection.** (A) Representative images (400X and 1000X) of apoptotic cells were marked with green fluorescence (TUNEL), the nuclei of cells are stained by blue fluorescence (DAPI), retina pigment epithelium are stained by red fluorescence (RPE65), and acquired by a fluorescence microscope. Arrowhead sign indicates apoptotic RPE signal. Scale bar= 20 μm and 50 μm.

**Figure S4: N-CM or H-CM administration attenuates the reduction of β-catenin in the retina following Aβ-injection**

Compared with the retinas of Sham+Veh rats, the retinas of the rats treated with Aβ displayed less abundant β-catenin (green, arrowhead) expression in the GCL and RPE. After the treatment with N-CM or H-CM in the presence of Aβ, the expression of β-catenin in the GCL and RPE were increased significantly.
