# Supplementary figures and images for: Mesenchymal stem cell‐conditioned medium attenuates the retinal pathology in amyloid‐β‐induced rat model of Alzheimer's disease: Underlying mechanisms
Source: Aging Cell. 2021 Mar 30;20(5):e13340. doi: 10.1111/acel.13340 (PMC8135003; doi:10.1111/acel.13340)

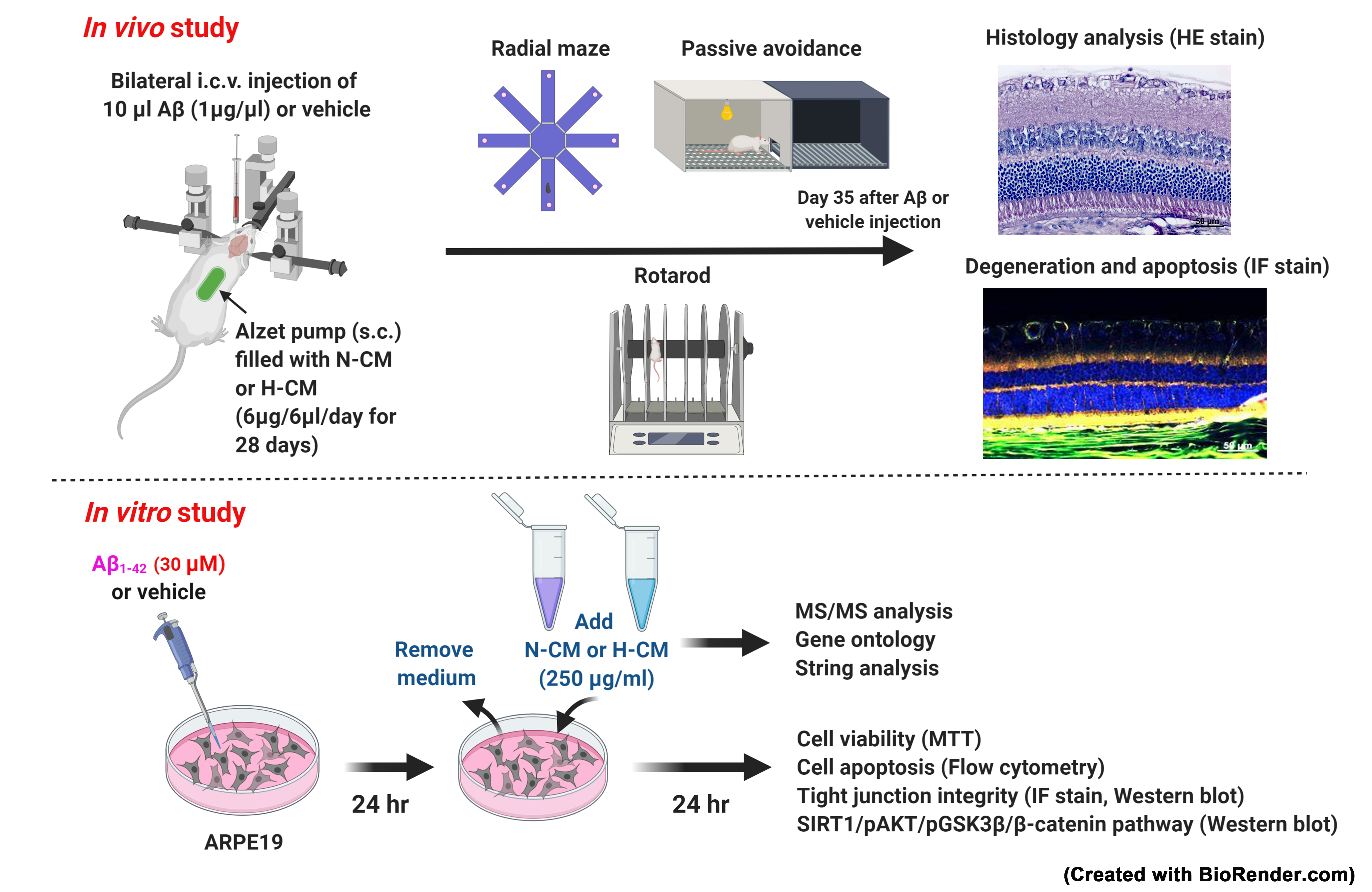

Supplement: Supplementary file 2 — Fig S1 [file ACEL-20-e13340-s004.tif]

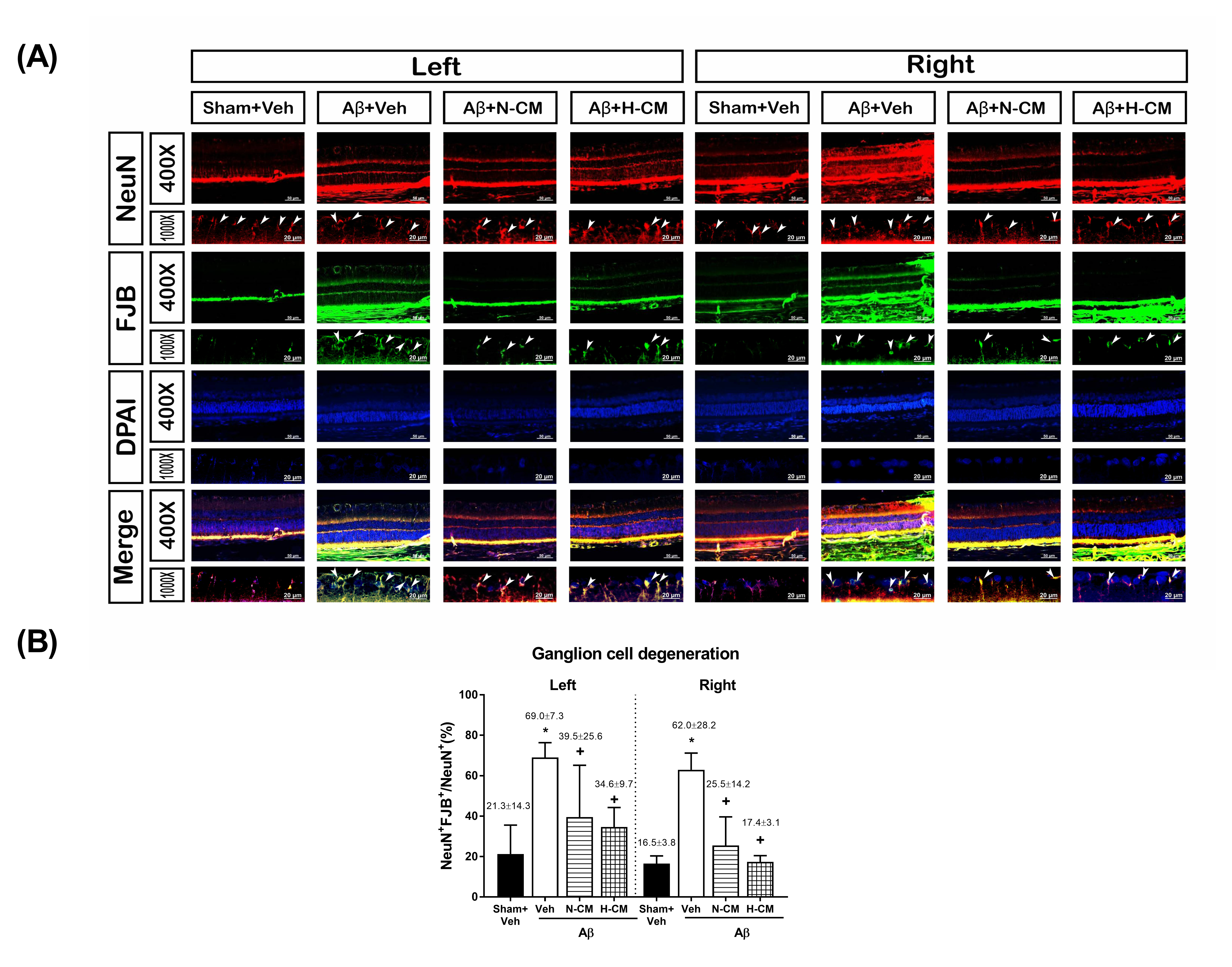

Supplement: Supplementary file 3 — Fig S2 [file ACEL-20-e13340-s001.tif]

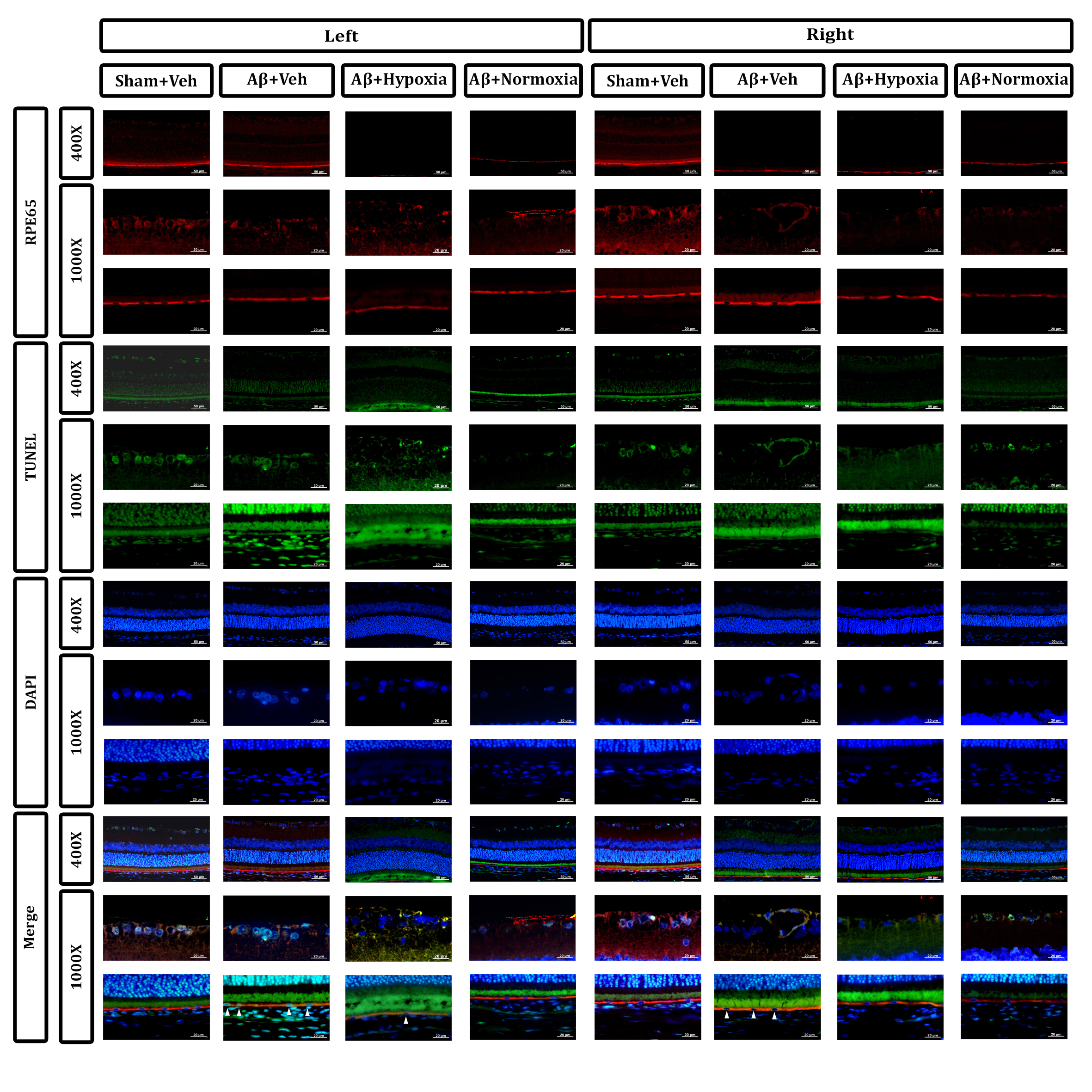

Supplement: Supplementary file 4 — Fig S3 [file ACEL-20-e13340-s005.tif]

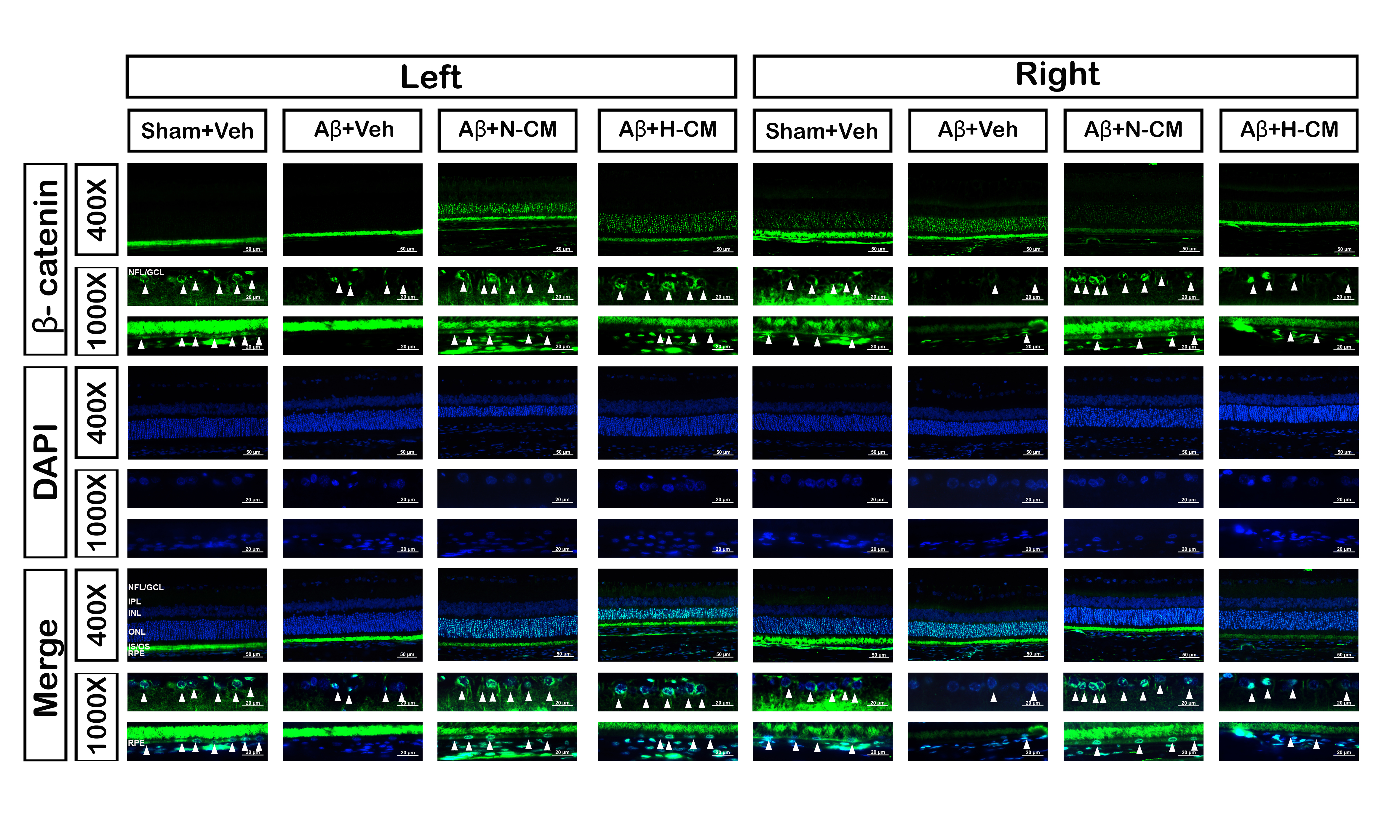

Supplement: Supplementary file 5 — Fig S4 [file ACEL-20-e13340-s002.tif]
